# *Jiawei Kongsheng Zhenzhong Pill* (JKZP) Alleviates Chronic Cerebral Hypoperfusion‐Induced Hippocampal Synaptic Damage via S100A10/tPA/BDNF Pathway

**DOI:** 10.1002/brb3.70328

**Published:** 2025-02-17

**Authors:** Qiaolan Wu, Yang Zhou, Chunxue Ou, Zu Gao, Xiaolin Wu, Yue Zhao, Yuan Wang, Zhichun Wu, Huayun Yu

**Affiliations:** ^1^ College of Traditional Chinese Medicine Shandong University of Traditional Chinese Medicine Jinan China; ^2^ Experimental Center Shandong University of Traditional Chinese Medicine Jinan China; ^3^ Shandong Provincial Co‐innovation Center of Classic Traditional Chinese Medicine Formula Jinan China

**Keywords:** *Jiawei Kongsheng Zhenzhong Pill* (JKZP), mild cognitive impairment (MCI), S100 calcium‐binding protein A10 (S100A10)/tissue‐type plasminogen activator (tPA)/brain‐derived neurotrophic factor (BDNF) pathway, synaptic plasticity

## Abstract

**Aims:**

To evaluate the effects of *Jiawei Kongsheng Zhenzhong Pill* (JKZP) on rats with ischemic mild cognitive impairment (MCI) and investigate the underlying mechanisms.

**Methods:**

The components of JKZP were analyzed using Q‐Orbitrap high‐resolution mass spectrometry (HRMS). The MCI rat model was prepared through gradual bilateral common carotid artery occlusion (BCCAO). The cognitive function, hippocampal pathological lesions, dendritic spine damage, synapse‐related, and S100 calcium‐binding protein A10 (S100A10)/tissue‐type plasminogen activator (tPA)/brain‐derived neurotrophic factor (BDNF) pathway‐associated molecules alterations were measured. Primary hippocampus neurons were subjected to oxygen‐glucose deprivation/reperfusion (OGD/R) injury, and JKZP‐containing serum was utilized for treatment. Lentiviral‐infected neurons were constructed with S100A10 knockdown using RNAi technology to investigate whether JKZP exerted its anti‐MCI effects via S100A10/tPA/BDNF pathway.

**Results:**

A total of 64 major components, including β‐asarone, ferulic acid, loganin, senkyunolide H, and cryptotanshinone, were identified by Q‐Orbitrap HRMS technology. JKZP had a notable impact on enhancing the cognitive abilities of rats with MCI. JKZP reduced the damage to the hippocampal CA1 region neuron and synaptic structure, reversed the decrease in dendritic spines, and increased the expressions of synapse‐associated proteins such as synaptophysin (SYN), growth‐associated protein 43 (GAP43), and postsynaptic density protein 95 (PSD95). Furthermore, JKZP treatment dramatically reduced the ratio of protein of BDNF (proBDNF)/mature BDNF (mBDNF) by activating S100A10/tPA, which was confirmed in primary hippocampus neurons in vitro. Moreover, sh‐S100A10 tremendously mitigated the inhibitory action of JKZP on OGD/R‐mediated synapse injury, decreased the activity of tPA, and thus improved the downstream pathway targets’ ratio, proBDNF/mBDNF.

**Conclusions:**

These results manifested that JKZP promoted neurological recovery after chronic cerebral ischemia by alleviating synaptic damage and activating the S100A10/tPA/BDNF pathway, thereby providing a novel perspective and a solid foundation against MCI.

## Graphical abstract

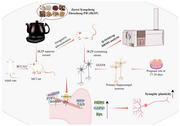

**Abbreviations**
BCCAO: bilateral common carotid artery occlusion
BDNF: brain‐derived neurotrophic factor
CCH: chronic cerebral hypoperfusion
GAP43: growth‐associated protein 43
JKZP: *Jiawei Kongsheng Zhenzhong Pill*.
mBDNF: mature BDNF
MCI: mild cognitive impairment
OGD/R: oxygen‐glucose deprivation/reperfusion
P75^NTR^
: P75 neurotrophic receptor
proBDNF: precursor BDNF
PSD95: postsynaptic density protein 95
S100A10: S100 calcium‐binding protein A10
SYN: synaptophysin
TCM: traditional Chinese medicine
tPA: tissue‐type plasminogen activator
TrkB: tropomyosin receptor kinase B
VCI: vascular cognitive impairment

## 1 Introduction

Vascular cognitive impairment (VCI), encompassing all vascular cognitive deficits such as mild cognitive impairment (MCI) and dementia, poses a serious threat to human physical and mental health and is becoming a major public health problem worldwide (Yan et al. 2021). MCI represents the preclinical and transitional stage between normal aging cognitive decline and dementia (Weil, Costantini, and Schrag 2018). A meta‐study revealed that the global prevalence of MCI in individuals aged 50 years and older was 15.56% (Bai et al. 2022), and the 2‐year cumulative incidence of dementia in patients with MCI aged 65 years and older was 14.9% (Petersen et al. 2018). MCI is considered to be a pivotal entry point to delay the progression of dementia. Therefore, early prevention, intervention, and therapeutic strategies are crucial.

Chronic cerebral hypoperfusion (CCH) is a fundamental mechanism underlying the onset and development of VCI. Risk factors such as hypertension, type 2 diabetes mellitus, and cerebral atherosclerosis cause a decrease in cerebral blood flow (de la Torre 2012; Rajeev et al. 2022), activating a cellular damage cascade that impairs synaptic activity within neuronal circuits and triggers neurodegenerative diseases related to motor, memory, and cognitive dysfunction (Yao et al. 2024). The hippocampus, especially the CA1 region, is the most ischemia‐sensitive functional region. After CCH, hippocampal neuronal signaling is impaired, manifesting as alterations in neuronal dendritic and synaptic morphology and density, synaptic plasticity impairments, synaptic loss, and decreased levels of synaptic‐associated proteins, then leading to cognitive decline and dementia (Li et al. 2023; Sekhon et al. 1997). Brain‐derived neurotrophic factor (BDNF) is considered a potential predictive biomarker of poststroke cognitive impairment, and the addition of serum BDNF to conventional prognostic factors may ameliorate cognitive impairment after ischemic stroke to some extent (Chang et al. 2024). It has been demonstrated that BDNF levels are closely related to synaptic function, and the precursor protein of BDNF (proBDNF) can be cleaved by protein hydrolysis to mature BDNF (mBDNF). proBDNF preferentially activates P75 neurotrophic receptor (P75^NTR^) to mediate apoptosis and exerts neurotoxic effects (Yang et al. 2021), whereas mBDNF selectively activates tropomyosin receptor kinase B (TrkB) to promote cell survival and synaptic remodeling (Wang et al. 2022). The proBDNF/mBDNF balance occupies a key role in the pathogenesis of cognitive impairment, with high proBDNF expression being a major factor causing MCI. Furthermore, proBDNF is regulated by the cleavage‐associated gene S100 calcium‐binding protein A10 (S100A10, also known as p11, nerve growth factor‐inducible protein 42) and endogenous tissue‐type plasminogen activator (tPA) production by native cells, especially in response to ischemic or other neuroinflammatory conditions, which can further mediate neuronal synaptic plasticity (Ludka et al. 2017; Park et al. 2016). Therefore, activating neuronal S100A10, modulating tPA activity, and intervening in proBDNF/mBDNF balance to improve synaptic structure and function may be promising therapeutic strategies for MCI intervention.

Recently, a growing number of studies have demonstrated that traditional Chinese medicine (TCM) treatment can reduce synaptic damage, increase synapse numbers, and improve the synaptic structure through multiple pathways, thereby alleviating cognitive impairment caused by CCH (Wang et al. 2019). According to TCM theory, MCI falls under the categories of “forgetfulness” and “dullness,” with the disease's location lying in the brain. The underlying cause is thought to be a deficiency of kidney essence, whereas stasis and blood stasis obstructing the orifices are outer phenomena. Moreover, tonifying the kidneys and activating the blood are the commonly used clinical treatment principle. *Jiawei Kongsheng Zhenzhong Pill* (JKZP) is derived from the *Kongsheng Zhenzhong Pill* in “Thousand‐Golden‐Prescriptions,” and *Salvia miltiorrhiza* Bunge, *Ligusticum sinense* “Chuanxiong,” *Cornus officinalis* Sieb. et Zucc., and *Cistanche deserticola* Wild were added to strengthen the efficacy in tonifying the kidneys, benefiting the vital essence, activating blood circulation, and removing blood stasis. Previous clinical trials have shown that JKZP can significantly improve cognitive function and quality of life in patients with vascular dementia (VaD) (Xu 2005). Pharmacological experiments have demonstrated that JKZP has anti‐neuronal apoptosis effects, promotes brain angiogenesis, enhances synaptic plasticity, and slows down the progression of cognitive damage in rats with CCH (Pang et al. 2016; Yu et al. 2016). Additionally, JKZP can mediate synaptic remodeling through the modulation of the Netrin‐1/DCC signaling pathway in poststroke depressed rats (Song 2022). However, the specific mechanism and the pharmacological basis of JKZP's effect on CCH‐induced synaptic damage and cognitive function remain to be further elucidated.

On the basis of the preceding information, the present study hypothesizes that JKZP can enhance neuronal synaptic plasticity and improve cognitive deficits caused by CCH by modulating proBDNF/mBDNF balance via S100A10/tPA. In our study, we employed the stepwise bilateral common carotid artery occlusion (BCCAO) method, which accurately simulates the pathophysiological process of long‐term cerebral insufficiency of cerebral perfusion (Tukacs et al. 2020), to construct an in vivo chronic cerebral ischemia rat model of MCI. Furthermore, in vitro experiments were conducted using the primary hippocampal neuronal oxygen‐glucose deprivation/reperfusion (OGD/R) model to simulate the in vivo cerebral ischemia effect (Xiao et al. 2023). Simultaneously, Q‐Orbitrap high‐resolution mass spectrometry (HRMS) was utilized to investigate the neuroprotective mechanisms and pharmacological basis of JKZP in MCI. The study aims to provide a reliable foundation for studying the mechanism of classical prescriptions as well as facilitating the transformation of TCM research results.

## 2 Materials and Methods

### 2.1 Reagents, Chemicals, and Antibodies

Neurobasal (Cat. 21103049), B27 Supplement 50X serum‐free (Cat. 17504044), GlutaMAX additive (Cat. 35050061), and fetal bovine serum (FBS, Cat. 10099‐141) were purchased from Thermo Fisher Scientific Inc., USA; trypsin (Cat. KGM25200) and penicillin‐streptomycin (Cat. KGY0023) were purchased from Keygen Biotech Co. Ltd., China; sugar‐free dulbecco's modified eagle medium (DMEM) (Cat. PM150271) was purchased from Pricella Biotech Co. Ltd., China; cell counting kit‐8 (CCK8, Cat. BS350B) was purchased from Biosharp Biotech Co. Ltd., China; Golgi kit (Cat. G1069) was purchased from Servicebio Biotech Co. Ltd., China; Triton X‐100 (Cat. T8200) and goat serum (SL038) were purchased from Solarbio Biotech Co. Ltd., China; enzyme‐linked immunosorbent assay (Elisa) kits (tPA, Cat. JL10999) were purchased from Jianglai Biotech Co. Ltd., China; RNA isolater Total RNA Extraction Reagent (Cat. R401‐01), HiScript III RT SuperMix for qPCR (+gDNA wiper) (Cat. R323‐01), and ChamQ Universal SYBR qPCR Master Mix (Cat. Q711‐02) were purchased from Vazyme Biotech Co. Ltd., China; BCA kit (Cat. SW101‐02) was purchased from Seven Biotech Co. Ltd., China; ECL chemiluminescence (Cat. ED0015‐B) was purchased from Sparkjade Biotech Co. Ltd., China; S100A10 RNAi lentivirus was supplied by GeneChem Technology Co. (Shanghai).

Primary antibodies including growth‐associated protein 43 (GAP43, Cat. ab75810), synaptophysin (SYN, Cat. ab32127), Neun (Cat. ab177487, Abcam), MAP2 (ab254264, Abcam), and GAPDH (Cat. ab8245, Abcam) were purchased from Abcam, USA; postsynaptic density protein 95 (PSD95, Cat. 12369‐1‐AP), tPA (Cat. 10147‐1‐AP), BDNF (Cat. 28205‐1‐AP), TrkB (Cat. 13129‐1‐AP), P75^NTR^ (Cat. 55014‐1‐AP), sortilin (Cat. 12369‐1‐AP), and β‐actin (Cat. HRP‐60008) were purchased from Proteintech Group Inc., China; S100A10 (Cat. 201226) was purchased from Zen‐Bio, China.

### 2.2 Animals

Two batches of animals were utilized in this study. For in vivo experiments, healthy male Wistar rats with a body mass of 250 ± 10 g (age 6–7 weeks) were employed, purchased from Beijing Viton Lihua Co. Ltd, with an animal production license No. SCXK (Beijing) 2016‐0006 and an animal quality certificate No. 110011211103958924. JKZP‐containing/blank serum preparation and primary hippocampal neuron culture experiments were performed using healthy male and female Sprague–Dawley (SD) rats with a body mass of 250 ± 10 g (age 6–7 weeks), purchased from Beijing Viton Lihua Co. Ltd, with an animal production license No. SCXK (Beijing) 2021‐0011, and an animal quality certificate No. 110011221110821826. All rats were housed in the SPF‐level animal room of the animal experiment center of Shandong University of TCM at a temperature of 19°C–27°C and a humidity of 40%–70%. The animals were free to ingest food and water and were acclimatized for 7 days prior to the experiments.

### 2.3 Preparation of JKZP

The Chinese medicine pieces of JKZP were purchased from Shandong Bokang TCM decoction Pieces Co. LTD, which were authenticated by Professor Hongyan Li and conformed to the specifications of the Pharmacopoeia of the People's Republic of China edition (2020). The extraction method and process of the water extract of JKZP have been described in detail in our previous study (Wu et al. 2024). *L. sinense* “Chuanxiong” and *Acorus tatarinowii* Schott were crushed and dipped in a distillation flask for 30 min, and the volatile oil was extracted by distillation for 6 h. The volatile oil was collected, and the filtrate and the dregs were stored temporarily. Then *Chinemys reevesii* (Gray) and *Os Draconis* (*Fossilia Ossia Mastodi*) were broken into pieces and decocted for 30 min and then added to other drugs and dregs to boil for 45 min. The second decoction was made, and the filtrate was combined and filtered. The filtrate was concentrated by spinning in a water bath at 65°C, and the volatile oils were combined. The extract was finally concentrated to 1.13 and 5.67 g/mL and stored at 4°C. The composition of JKZP is shown in Table 1.

**TABLE 1:** The composition of *Jiawei Kongsheng Zhenzhong Pill* (JKZP).

Latin name	Chinese name	Part used	Grams (g)	Cat.
*Chinemys reevesii* (Gray)	Guijia	Shell	18	19081001
*Os Draconis* (*Fossilia Ossia Mastodi*)	Longgu	Bone fossils of mammals	18	20200201
*Salvia miltiorrhiza* Bunge	Danshen	Root and rhizome	15	20121401
*Cornus officinalis* Sieb. et Zucc.	Shanzhuyu	Sarcocarp	15	20010801
*Ligusticum sinense* “Chuanxiong”	Chuanxiong	Rhizome	12	20092103
*Cistanche deserticola* Wild	Roucongrou	Succulent stem	12	20121904
*Acorus tatarinowii* Schott	Shichangpu	Root	9	20103001
*Polygalae tenuifolia* Willd	Yuanzhi	Root	9	20113001

### 2.4 Preparation of JKZP‐Containing Serum and Lyophilized Powder

Thirty SD male rats were randomly divided into the JKZP‐containing serum group (20, gavaged with 56.7 g/kg of JKZP at 5 times the equivalent clinical dose) and blank serum group (10, gavaged with equal volume of saline). The method of preparation of JKZP‐containing serum has been reported in a previous study (Wu et al. 2024). Blood was collected from the abdominal aorta of rats, centrifuged, and the serum was collected. The complement was inactivated in a constant temperature water bath at 56°C for 30 min, filtered through a 0.22 µm cell strainer to remove bacteria, partitioned, and placed at −80°C. The serum was frozen at −80°C overnight, then dried in a vacuum desiccator (FD‐1A‐50 Lophilizer, Pogson) for 3 days. The lyophilized powder was collected, and the serum lyophilized powder master batch was prepared at a concentration of 100% using neuron complete medium, filtered, and sterilized by 0.22 µm cellular filters, partitioned, and stored at −20°C.

### 2.5 Q‐Orbitrap HRMS Analysis of JKZP

JKZP aqueous extract, drug‐containing serum, and blank serum samples were respectively mixed with 1 mL of 80% methanol, vortexed, and centrifuged at 4°C for 10 min with a centrifugal force of 20,000 × *g*, and the supernatant was filtered through a 0.22 µm membrane. The samples were analyzed by Q‐Orbitrap HRMS system (Ultimate 3000 RS ultra‐high performance liquid chromatograph, Q Exactive high‐resolution mass spectrometer, Thermo Fisher Scientific, USA). XB‐C18 columns (250 × 4.6 mm^2^, 5 um, Welch) were used. The mobile phases were Solvent A (aqueous phase, 0.1% formic acid aqueous solution) and Solvent B (organic phase, 0.1% formic acid acetonitrile). The flow rate was 1.20 mL/min, the column temperature was set at 35°C, the autosampler temperature was 10.0°C, and the autosampler injection volume was 5.00 µL. Q‐Orbitrap‐MS was performed using an electrospray ionization source (ESI) with positive and negative ion mode detection. Argon was used to stabilize the spray, and the scanning mode was Full mass/dd‐MS2. Identification of the test compounds was performed by retention time, precise relative molecular mass, and secondary mass spectral fragmentation. The chromatographic gradients are provided in Table 2.

**TABLE 2:** The chromatographic gradients.

Time/min	aqueous phase/%	aqueous phase/%
0.0	98	2
1	98	2
5	80	20
10	50	50
15	20	80
20	5	95
25	5	95
26	98	2
30	98	2

### 2.6 Animal Models and Treatments

**FIGURE 1:**
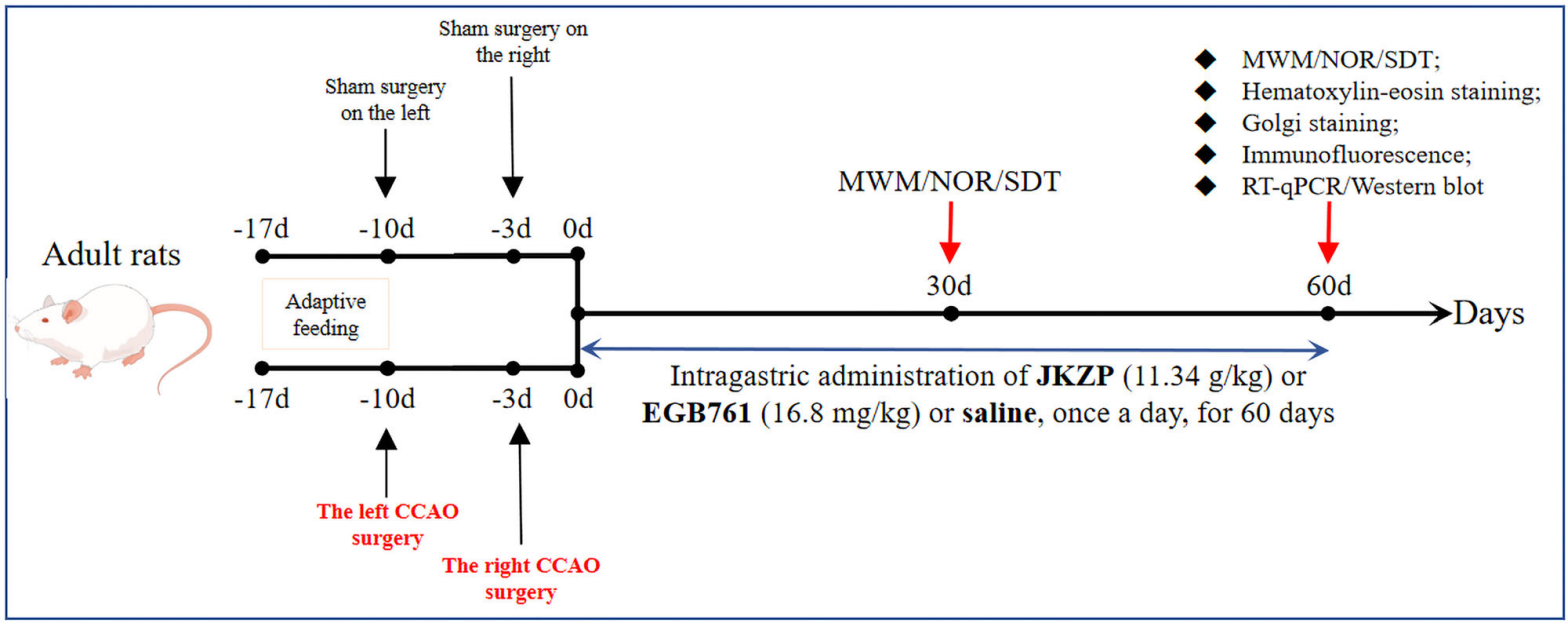
The in vivo experimental process. *Note*: −17 days are the time when the rats were purchased. The BCCAO surgery was started after 7 days of adaptive feeding. JKZP, *Jiawei Kongsheng Zhenzhong Pill*; MWM, Morris water maze; NOR, novel object recognition; SDT, step‐down test.

In our earlier study and preexperiment, we found that clinically equivalent doses of JKZP could better improve the neurological deficit symptoms in MCI rats (Figure S1). It has also been proven to be effective and economical (Pang et al. 2016; Yu et al. 2016). A total of 85 Wistar male rats were fasted for 12 h before surgery and randomly divided into a sham operation group (Sham, 20) and a model group (MCI, 65). The rat model with MCI was prepared by stepwise BCCAO (Tukacs et al. 2020). Continuous inhalation of 3% isoflurane was used to maintain anesthesia and ligated the left common carotid artery (CCA). The incision was sutured, and 200,000 units of penicillin sodium were intraperitoneally injected after the operation for 3 consecutive days to prevent infection. The sham‐operated group underwent the same procedure without arterial ligation. One week later, the right CCA was ligated using the same method. Two rats died during the operation. The remaining MCI rats were randomly divided into three groups: the JKZP group (JKZP, 11.34 g/kg of JKZP by gavage at an equivalent clinical dose), the *Ginkgo biloba* extract group (EGB761, 16.8 mg/kg of *G. biloba* leaf extract by gavage at an equivalent clinical dose), and the model group (Model), with 21 rats in each group and 20 rats in the Sham group. Equivalent doses of saline were administered by gavage in the model and sham groups. EGB761 (tablet, 40 mg, Cat. H20140768) was prepared as 1.68 mg/mL suspension with saline. The rats in each group were gavaged once a day at 10 AM every day for 60 days. Body mass was weighed once a week, and the dose was adjusted according to the changes in body mass. Behavioral tests were performed on the 30th and 60th days of the intervention. The in vivo experimental process is illustrated in Figure 1.

### 2.7 Morris Water Maze (MWM)

MWM was observed for spatial learning and memory abilities. A hidden platform was placed in the center of quadrant 1 of a 1.6 m diameter pool. The animals were introduced into the water from different quadrants and underwent training 4 times a day over 4 consecutive days. The time taken by the rats to climb onto the platform within 60 s was recorded as the latency to the target. On the fifth day, the platform was removed, and the number of times the rats crossed the platform and the trajectory of the rats within 60 s was recorded.

### 2.8 Novel Object Recognition (NOR) Test

NOR was assessed for recognition and memory abilities. The rats were placed alone in the open box to explore for 5 min on the first day (adaptation experiment), alone in the open box with two identical objects on the second day (familiarization experiment), and alone in the open box with two different objects on the third day, and the time spent exploring each object was recorded (test experiment). The time spent exploring the new object (T1) and the time spent on the old object (T2) were recorded. The recognition and discrimination indexes were used to evaluate the rats' recognition ability.

$$\mathrm{Recognition}\;\mathrm{index}=\left(T1-T2\right)/\left(T1+T2\right)\times 100\%$$

$$\mathrm{Discrimination}\;\mathrm{index}=T1/\left(T1+T2\right)\times 100\%$$

### 2.9 Step‐Down Test (SDT)

SDT was conducted to evaluate passive avoidance learning and memory ability. The rats were placed in the jumping box to adapt for a 4‐min adaptation period. After 24 h, the rats were placed on the safety platform again. The time required for the rats to jump from the platform within 5 min and the number of times they jumped off the platform during the recording period (i.e., the error times) was observed. If the animals did not jump off the platform during the observation period, the error times were recorded as 0 times.

### 2.10 Hematoxylin‐Eosin Staining (HE)

At the end of the intervention, rats were anesthetized by continuous inhalation of 3% isoflurane. After perfusion with saline and 4% paraformaldehyde via cardiac perfusion, the brain tissues were collected and placed in a 4% paraformaldehyde solution for fixation. The tissues were then dehydrated, embedded in dipping wax, sliced, stained with HE, dehydrated by gradient dehydration, and sealed. The morphology of the hippocampus was observed under a light microscope and photographed.

### 2.11 Golgi Staining

Rat hippocampal tissue was cut into 2–3 mm small pieces, rinsed, and stained using a Golgi kit (Servicebio, G1069). The tissue was then sectioned, treated sequentially with concentrated ammonia and acidic firm film fixative, and sealed with glycerol gelatin. The CA1 region of the hippocampus was localized under a light microscope at 10 × magnification, and the dendritic spines of the hippocampus were observed and analyzed under 1000 × magnification. Image J was used to count the number of dendritic spines in the intercepted dendritic spine images, and then the number of dendritic spines/dendritic length (5 µm) was used to obtain the spine density (Spines/5 µm).

### 2.12 Transmission Electron Microscopy

Hippocampal tissues were cut into pieces and placed in 2.5% glutaraldehyde paraformaldehyde phosphate buffer for 4°C fixation for 4 h, washed with phosphate buffer solution (PBS) for 15 min × 3 times, and 1% osmium tetroxide for 4°C fixation for 2 h, washed with PBS for 15 min × 3 times. Gradient dehydration of 50%, 70%, 80%, and 90% acetone was carried out for 15 min each, and 100% acetone dehydration was carried out for 15 min × 2 times. The samples were localized under a light microscope and photographed under a transmission electron microscope.

### 2.13 Immunofluorescence for Rats’ Paraffin Section

Paraffin sections were deparaffinized to water, antigenically repaired, blocked, and then incubated with drops of the appropriate primary antibody in a humid box at room temperature for 1 h. The corresponding secondary antibody was added and incubated for 1 h. Opal dye (1:100) was added to cover the tissues and incubated at room temperature for 10 min. Subsequently, DAPI (1:100) was added to stain the cell nuclei for 5 min. The sections were sealed with an anti‐fade mounting medium and immediately observed and photographed under a fluorescence microscope. Image J software was utilized for semi‐quantitative and quantitative analyses. The primary antibodies used were as follows: GAP43 (1:500), SYN (1:400), PSD95 (1:100), S100A10 (1:100), tPA (1:100), Neun (1:200). Opal 520 (OP‐001001), Opal 570 (OP‐001003), Opal 620 (OP‐001004), and Opal 690 (OP‐001006) were at 1:100 dilution rate.

### 2.14 Primary Hippocampal Neuron Culture and OGD/R Model Preparation

Forty SD rats (5 males and 35 females) were caged together (1 male and 2 females in a cage) in batches according to the experimental requirements. Pregnant rats at 17–18 days of gestation were euthanized and sterilized. Fetal rat hippocampal tissue was isolated, clipped, and digested using trypsin. Cells were then filtered, centrifuged, resuspended, and re‐filtered through 45 µm sterile cell filters and counted. The cells were then inoculated into poly‐d‐lysine coated culture flasks/plates. After 4 h, the medium was changed to neuronal complete medium, which consists of 96% Neurobasal, 2% B27 Supplement 50X serum‐free, 1% GlutaMAX, and 1% penicillin‐streptomycin. Subsequently, the medium was changed every 3 days. Cells were incubated until day 7 for intervention (Figure 7A) with greater than 95% purity (Figure 7B). For the OGD/R model, the original medium was discarded, and neurons were washed twice with pre‐warmed PBS and then added to pre‐warmed sugar‐free DMEM medium. The neurons were placed in a triple‐gas incubator (Forma steri‐cycle i160, Thermo) (1% O_2_, 94% N_2_, 5% CO_2_, and 37°C) for OGD modeling. The CCK8 method was used to determine that the optimal OGD modeling time was 0.5 h, resulting in a survival rate of approximately 50% (Figure 7C).

### 2.15 Cell Viability and Cytotoxicity Assays

Cells were inoculated in 96‐well plates (3 × 10^4 cells/well). At the end of the intervention, each well was incubated with 100 µL of medium containing 10% CCK8 for 2 h. The absorbance was detected at 450 nm on a microplate reader. Cell viability was calculated as follows: cell viability (%) = OD (experimental group−blank control group)/OD (normal group−blank control group) × 100%.

### 2.16 Cell Immunofluorescence

Cells (2 × 10^5 cells/well) were inoculated on the coverslips in 24‐well plates. At the end of the intervention, cells were fixed with 4% paraformaldehyde for 20 min at room temperature. Non‐cellular membrane proteins were required to be incubated with 0.1% Triton X‐100 for 20 min at room temperature. Then, 10% goat serum was used to block for 30 min. The primary antibody was incubated overnight at 4°C protected from light. The following day, a secondary antibody was added and incubated at room temperature for 1 h. The slices were sealed with a DAPI‐containing anti‐fade mounting medium. Images were obtained using a fluorescence microscope. The primary antibodies included MAP2 1:1000, SYN 1:500, GAP43 1:500, and PSD95 1:200.

### 2.17 Enzyme‐Linked Immunosorbent Assay (ELISA)

The cell supernatant was collected and centrifuged at 12,000 rpm/min for 5 min at 4°C (*r* = 7 cm). According to the instructions of S100A10 and tPA kits, the samples were spiked, incubated with the biotinylated antibody for 1 h at 37°C away from light, then reacted with enzyme conjugate for 30 min, and finally reacted with a substrate for 15 min. The reaction was terminated by adding a termination solution, and the OD value was measured at 450 nm.

### 2.18 RT‐qPCR Assay

Total RNA from hippocampal tissues and cells was extracted using the Trizol method. Reverse transcription and amplification were subsequently performed. Primers were synthesized by Accurate Biotechnology (Hunan) Co. Ltd., and the primer sequences are shown in Table 3. Using GAPDH or β‐actin as the internal reference gene, the mRNA expression levels of S100A10, mBDNF, P75^NTR,^ and TrkB were measured.

**TABLE 3:** Primer sequences.

Gene	Primer sequences (5′ → 3′)		Length (BP)
S100A10	F	CTTGACAAAGGAGGACCTGAGAGT	128
	R	CCACTTTTCCATCTCGGCACTG	
mBDNF	F	AATAATGTCTGACCCCAGTGCC	140
	R	GCGGTTTCCTTCTCCAAGCC	
P75^NTR^	F	TACAGTGGCGGATATGGT	94
	R	GGAGCAATAGACAGGAATGA	
TrkB	F	TGAGCTGAACTCCTGGGACTA	119
	R	GTCACAGCTCACAACAAGCAG	
GAPDH	F	AAGGAGTAAGAAACCCTGGACC	135
	R	GTCTGGGATGGAATTGTGAGG	
β‐Actin	F	GAGATTACTGCCCTGGCTCCTA	148
	R	ACTCATCGTACTCCTGCTTGCTG	

Abbreviations: mBDNF, mature BDNF; P75^NTR^, P75 neurotrophic receptor; S100A10, S100 calcium‐binding protein A10; TrkB, tropomyosin receptor kinase B.

### 2.19 Western Blotting (WB)

Proteins were extracted from hippocampal tissues or primary hippocampal neurons and then quantified by a BCA kit. Proteins were separated by SDS‐PAGE and then transferred to PVDF membranes, which were blocked with 5% skimmed milk powder for 2 h at room temperature. The membranes were incubated with the primary antibody at 4°C overnight. The corresponding secondary antibodies were incubated at room temperature for 1 h the following day. Finally, the PVDF membranes were developed utilizing ECL chemiluminescence, and the results were analyzed quantitatively. The primary antibodies included PSD95 (1:1000), SYN (1:5000), GAP43 (1:1000), S100A10 (1:750), tPA (1:1000), BDNF (1:1000), TrkB (1:1000), P75^NTR^ (1:1000), sortilin (1:1000), GAPDH (1:10000), and β‐actin (1:20000).

### 2.20 Lentivirus Transfection and Cell Grouping

Lentiviral short hairpin RNA (shRNA), supplied by Genechem Co. Ltd. (Shanghai, China), was used to stably silence the expression of rat S100A10. Primary hippocampal neurons cultured until day 4 were transfected by lentivirus with different multiplicities of infection (MOI) of 20, 40, and 80. The lentivirus carrying GFP fluorescence expression was observed under the microscope after 72 h, and the knockdown effect was verified. The MOI value of 80 was ultimately screened (Figure 8A), and the most suitable fragment, sh‐3 (Figure 8B), was selected for subsequent experiments. Primary neurons were divided into the following groups: Normal group (Normal), OGD/R group (OGD 0.5 h), JKZP group (OGD 0.5 h, 5% JKZP‐containing serum), sh‐NC group (OGD 0.5 h, blank viral infection for 72 h), sh‐S100A10 group (OGD 0.5 h, sh‐S100A10 lentivirus infection for 72 h), and sh‐S100A10 plus JKZP group (OGD 0.5 h, sh‐S100A10 lentivirus infection for 72 h, 5% JKZP‐containing serum, abbreviated as sh + JKZP). ICC, ELISA, RT‐qPCR, and WB assays were performed at the end.

### 2.21 Statistical Analysis

Statistical analyses were conducted using GraphPad Prism software (v. 8.0, GraphPad Software, Inc., LaJolla, CA, USA). All results were repeated at least three times and were presented as mean ± standard deviation. One‐way analysis of variance (ANOVA) was used for comparisons between multiple groups, whereas the least significant difference method *LSD*‐*t*‐test was used for two‐by‐two comparisons between groups, with *p *< 0.05 indicating that the difference was statistically significant.

## 3 Results

### 3.1 Q‐Orbitrap HRMS Results of JKZP

**FIGURE 2:**
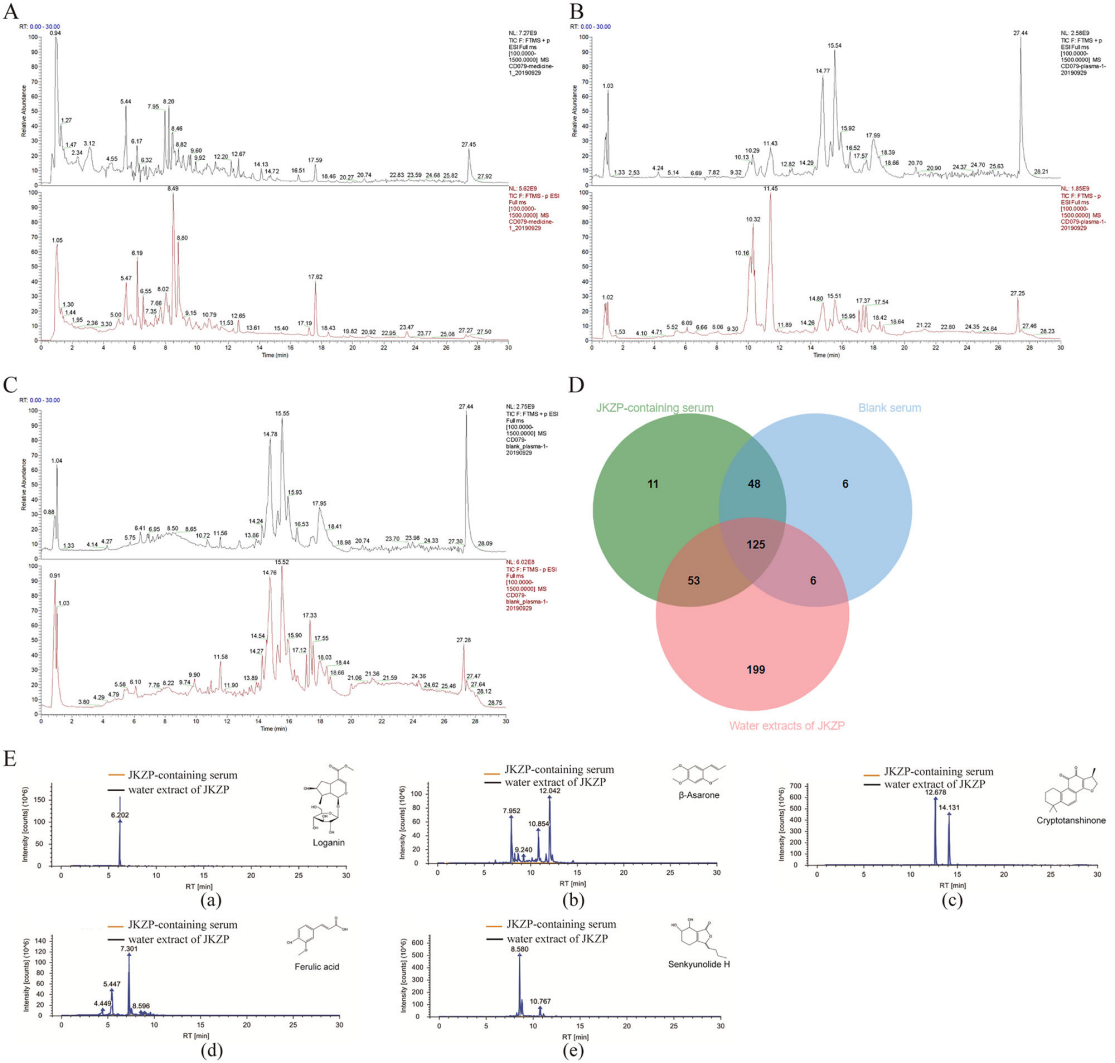
Q‐Orbitrap HRMS results of JKZP. (A–C) Total ion flow diagram of JKZP aqueous extract, JKZP‐containing serum and blank serum; (D) the components in JKZP aqueous extract, JKZP‐containing serum and blank serum were intersected (mzCloud Best Match ≥60); (E) Sample quality control: (a) Loganin; (b) β‐asarone; (c) cryptotanshinone; (d) ferulic acid; (e) senkyunolide H. Note: The A–C total ion flow diagram has positive ion mode in black in column 1 and negative ion mode in red in column 2. JKZP, *Jiawei Kongsheng Zhenzhong Pill*.

Total ionograms of JKZP aqueous extract, drug‐containing serum, and blank serum were collected in both positive and negative ion modes (Figure 2A–C). The data collected by high‐resolution liquid chromatography were initially processed by CD2.1 (Thermo Fisher), then searched in databases (mzCloud, mzVault, and ChemSpider), and compared with the relevant literature to identify the ion peaks in the total ionograms of the samples. An integrated score of ≥60 in the mzCloud (the higher the value, the higher the confidence of the identified results) was matched a total of 383 compounds in the JKZP aqueous extract (Table S1), 237 compounds in the JKZP‐containing serum, and 185 in the blank serum. Comparative analysis with rat blank serum identified a total of 64 main blood entry components/metabolic components of JKZP (Table S2), of which 53 overlapped with the components of the JKZP aqueous decoction (Table S3, Figure 2D). These mainly included phenolic acids, iridoid glycosides, phthalides, and diterpene quinones. According to the Pharmacopoeia of the People's Republic of China (2020), the key components of this formula include loganin, β‐asarone, cryptotanshinone, ferulic acid, and senkyunolide H, which can be used for quality control of the formula (Figure 2E, Table 4).

**TABLE 4:** Representative constituents in *Jiawei Kongsheng Zhenzhong Pill* (JKZP) aqueous extract and JKZP‐containing serum.

No.	Rt (min)	Formula	Molecular weight	Identification	Derived from
a	6.21	C_17_H_26_O_10_	407.17848	Loganin	*Cornus officinalis* Sieb. et Zucc.
b	7.939	C_12_H_16_O_3_	208.10936	β‐Asarone	*Acorus tatarinowii* Schott and *Ligusticum sinense* “Chuanxiong”
c	14.143	C_19_H_20_O_3_	296.13995	Cryptotanshinone	*Salvia miltiorrhiza* Bunge
d	7.138	C_10_H_10_O_4_	194.05691	Ferulic acid	*Ligusticum sinense* “Chuanxiong” and *Salvia miltiorrhiza* Bunge
e	8.58	C_12_H_16_O_4_	206.09315	Senkyunolide H	*Ligusticum sinense* “Chuanxiong”

### 3.2 JKZP Ameliorates Learning, Memory, and Cognition Impairment in MCI Rats

**FIGURE 3:**
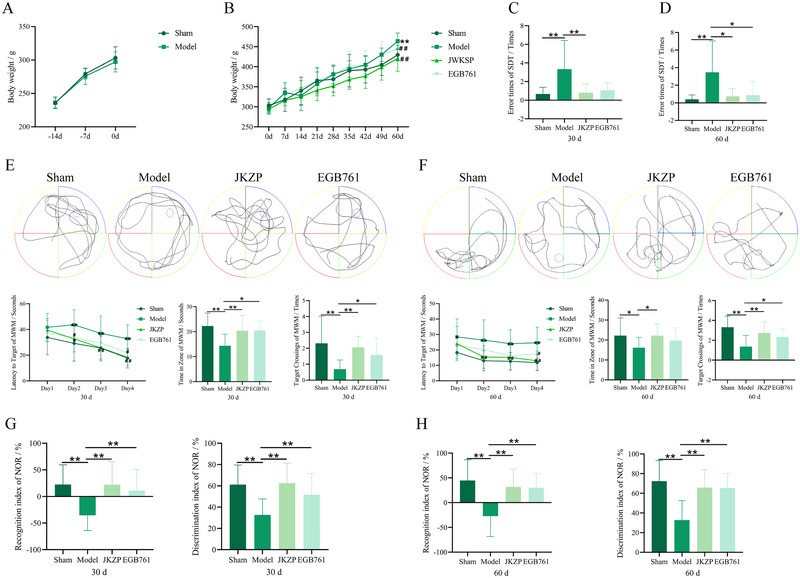
JKZP improved the learning, memory, and cognitive ability of MCI rats. (A) Body weight change of rats after modeling (Sham *n* = 19, Model *n* = 57); (B) body weight change of rats in each group after grouping (*n* = 19); (C) SDT of 30 days (*n* = 15); (D) SDT of 60 days (*n* = 15); (E) MWM of 30 days (*n* = 19); (F) MWM of 60 days (*n* = 19); (G) NOR test of 30 days (*n* = 19); (H) NOR test of 60 days (*n* = 19). *p < 0.05, **p < 0.01. JKZP, *Jiawei Kongsheng Zhenzhong Pill*.

As shown in Figure 3A,B, there was no significant difference in rats’ body weight before intervention (*p *> 0.05). During the observation period, the rats in all groups showed a gradual increase in body weight. To assess the effects of JKZP on the learning, memory, and cognitive ability of MCI rats, the SDT, MWM, and NOR experiments were conducted. At 30 and 60 days of drug administration, compared with the sham group, rats in the model group showed a significant increase in the error times of SDT (*p *< 0.01) (Figure 3C,D), a significant prolongation of the latency to target on the second day of training in the MWM test (*p *< 0.01), a significant decrease in the time spent in the target zone and the times of crossing the platform (*p *< 0.01) (Figure 3E,F), and a significant decrease in the recognition and discrimination indexes of NOR experiment (*p *< 0.01) (Figure 3G,H). However, compared with the model group, rats in the JKZP and EGB761 groups had significantly fewer errors in the SDT (*p *< 0.05, *p *< 0.01) (Figure 3C,D), shorter latency to target (*p *< 0.05, *p* < 0.01), more time in the target zone, and more times target crossing the platform (*p* < 0.05, *p* < 0.01) (Figure 3E,F). Additionally, they reversed the decline in the recognition and discrimination indexes in the NOR experiments (*p *< 0.01) (Figure 3G,H). These indicate that JKZP ameliorates learning, memory, and cognition impairment in rats with MCI.

### 3.3 JKZP Reduces Damage in the Hippocampal

**FIGURE 4:**
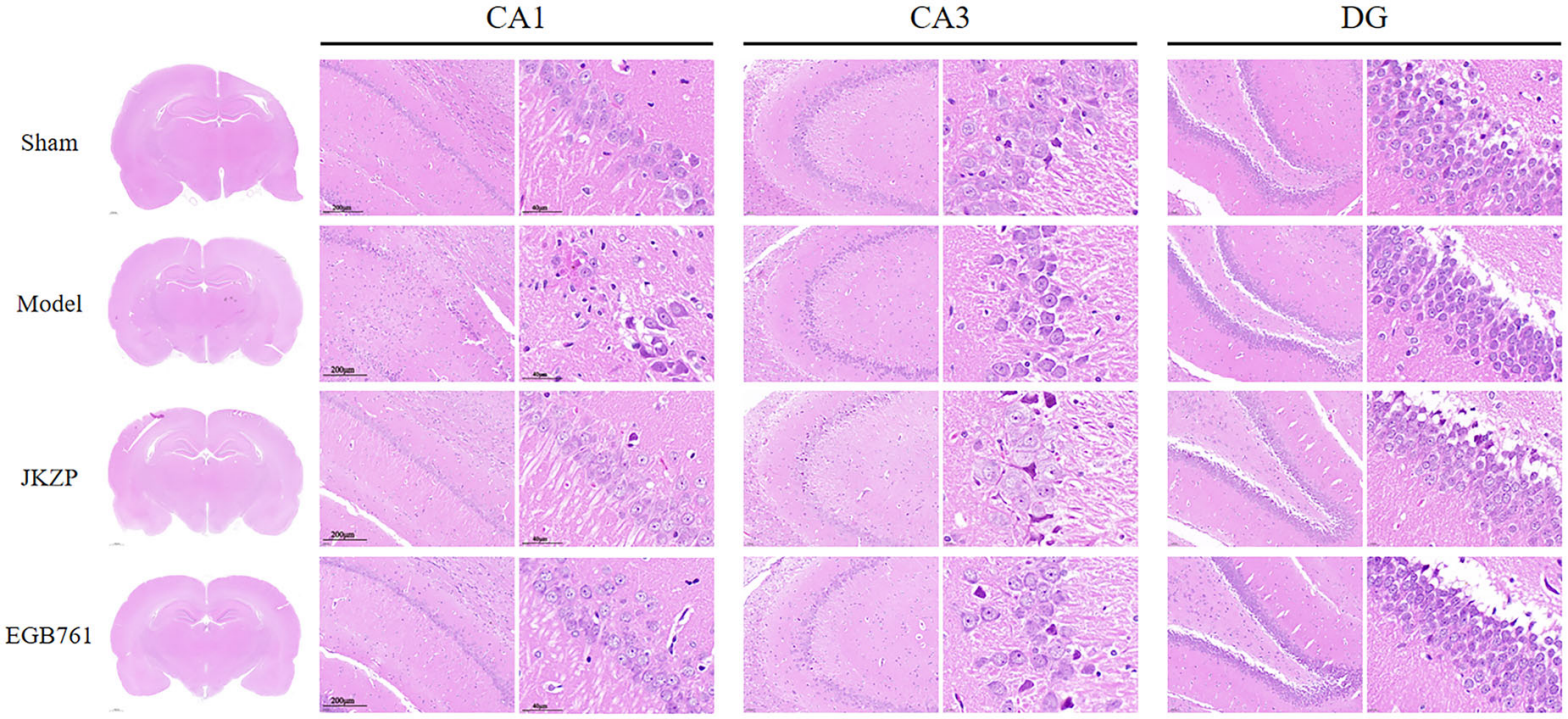
JKZP alleviated pathologic morphology in the hippocampal of MCI rats (HE, ×200, ×1000. *n* = 3).

HE staining was used to assess the histopathological morphology of CA1, CA3, and DG regions in the hippocampus. The hippocampus, especially the CA1 area, is the most sensitive functional area to ischemia. Neurons in the CA1 area are more anatomically complete, with a clear structure and reduced intermingling of visualization with other neurons (Kim et al. 2024), making the CA1 area ideal for observation of pharmacological effects of JZKP. In the model group, the number of hippocampal neurons was decreased, the arrangement was sparse and disordered, and the nucleus showed loose chromatin, pyknosis, or disappearance of karyolysis, especially the CA1 region. However, in the JKZP and EGB761 groups, the damage was somewhat improved, with the cellular morphology and arrangement being more regular than that of the model group and reduced nucleolysis (Figure 4). Therefore, in the following in vivo experimental studies, we will focus on the CA1 region.

### 3.4 JZKP Improves Synaptic Plasticity in Hippocampal Neurons of MCI Rats

**FIGURE 5:**
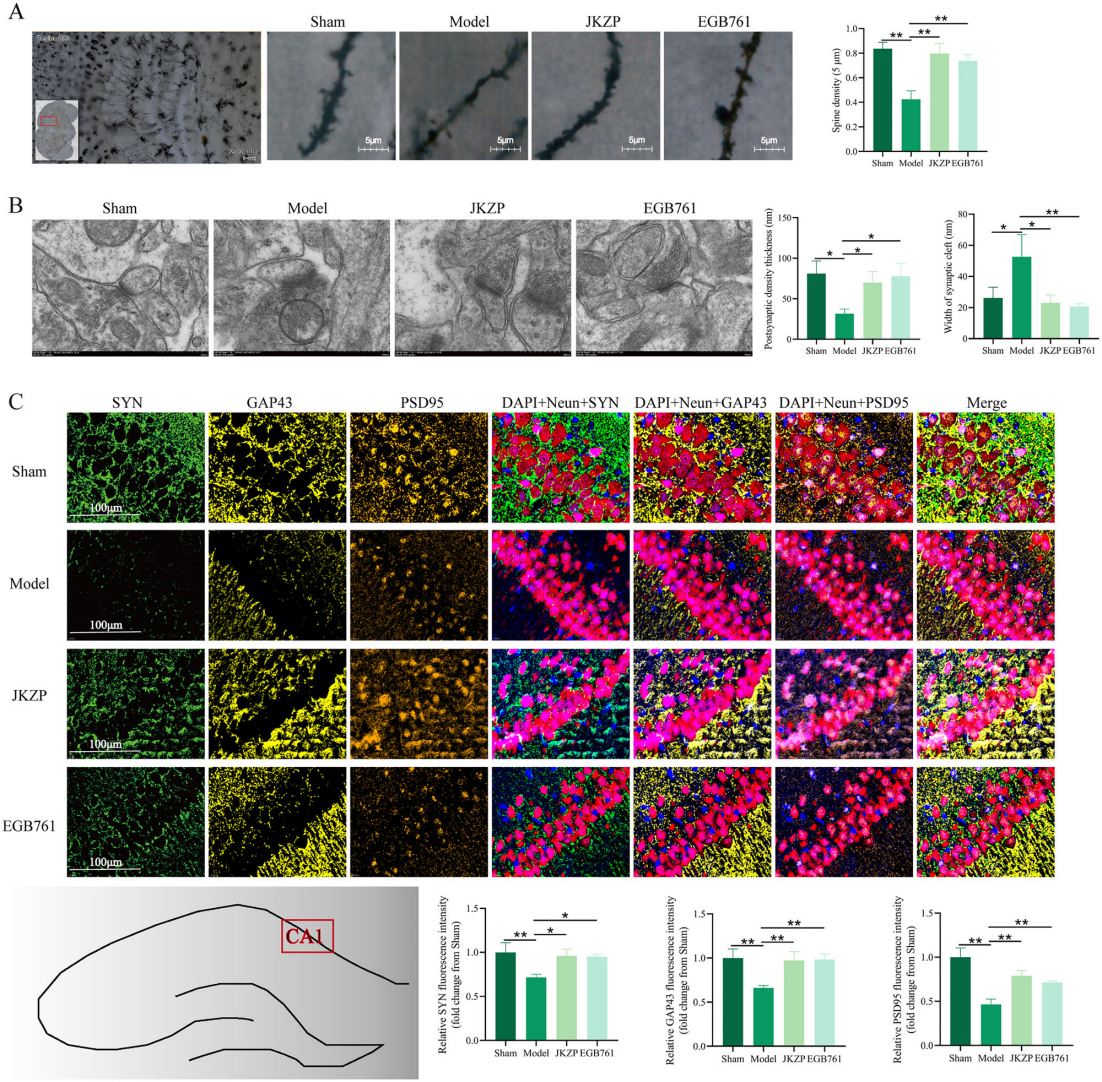
JKZP improves synaptic plasticity of hippocampal neurons in MCI rats. (A) Golgi staining shows the density of dendritic spines of CA1 region hippocampal neurons (*n* = 3, ×1000); (B) transmission electron microscopy was used to observe the synaptic ultrastructure of hippocampal neurons (*n* = 3, ×30.0 k). (C) Immunofluorescence observation on the co‐expression of PSD95, GAP43, and SYN in the hippocampal CA1 area with neuronal markers Neun (*n* = 3, ×1000, 620 orange‐PSD95, 570 yellow ‐GAP43, 520 green‐SYN, 690 red‐Neun).

Golgi staining was employed to observe the morphology of dendritic spines. As displayed in Figure 5A, the dendritic spines of hippocampal neurons in the sham group exhibited relatively regular morphology, with a neat arrangement and high abundance. The number of mushroom‐shaped dendritic spines was also relatively high. Compared with the sham group, dendritic spines in the model group exhibited reduced branching, irregular morphology, loose arrangement, and a sparse number (*p *< 0.01). Compared with the model group, dendritic spines in the JKZP and EGB761 groups showed regular morphology, a neat arrangement, and an increased number of mushroom‐like spines (*p *< 0.01).

Transmission electron microscopy revealed the synaptic ultrastructure in Figure 5B. In the sham group, the synaptic architecture was intact, with a distinctly visible synaptic cleft, well‐defined pre‐synaptic and post‐synaptic membranes, a uniformly thickened post‐synaptic membrane, and an abundance of uniformly distributed synaptic vesicles. Although in the model group, the pre‐synaptic membrane, post‐synaptic membrane, and synaptic cleft were fused and indistinguishable, the post‐synaptic membrane density was diminished (*p *< 0.05), and the width of the synaptic cleft was incremental (*p *< 0.05). Compared with the model group, in the JKZP and EGB761 groups, the synaptic structure appeared relatively normal, exhibiting a clearer synaptic cleft, increased post‐synaptic membrane density, and reduced width of synaptic cleft (*p *< 0.05, *p *< 0.01).

Immunofluorescence was utilized to detect the expression of synapse‐related proteins in neurons within the CA1 region of the hippocampus. As shown in Figure 5C, the fluorescence intensity of proteins SYN, GAP43, and PSD95 was significantly reduced in the model group compared with the sham group (*p *< 0.01). Both JKZP and EGB761 significantly reversed the reduction in synapse‐associated proteins compared with the model group (*p *< 0.05, *p *< 0.01). These results suggest that JKZP improves synaptic plasticity in neurons within the hippocampal CA1 region. These results indicated that JKZP could improve synaptic plasticity in hippocampal neurons of MCI rats.

### 3.5 Effects of JKZP on the S100A10/tPA/BDNF Pathway in MCI Rats

**FIGURE 6:**
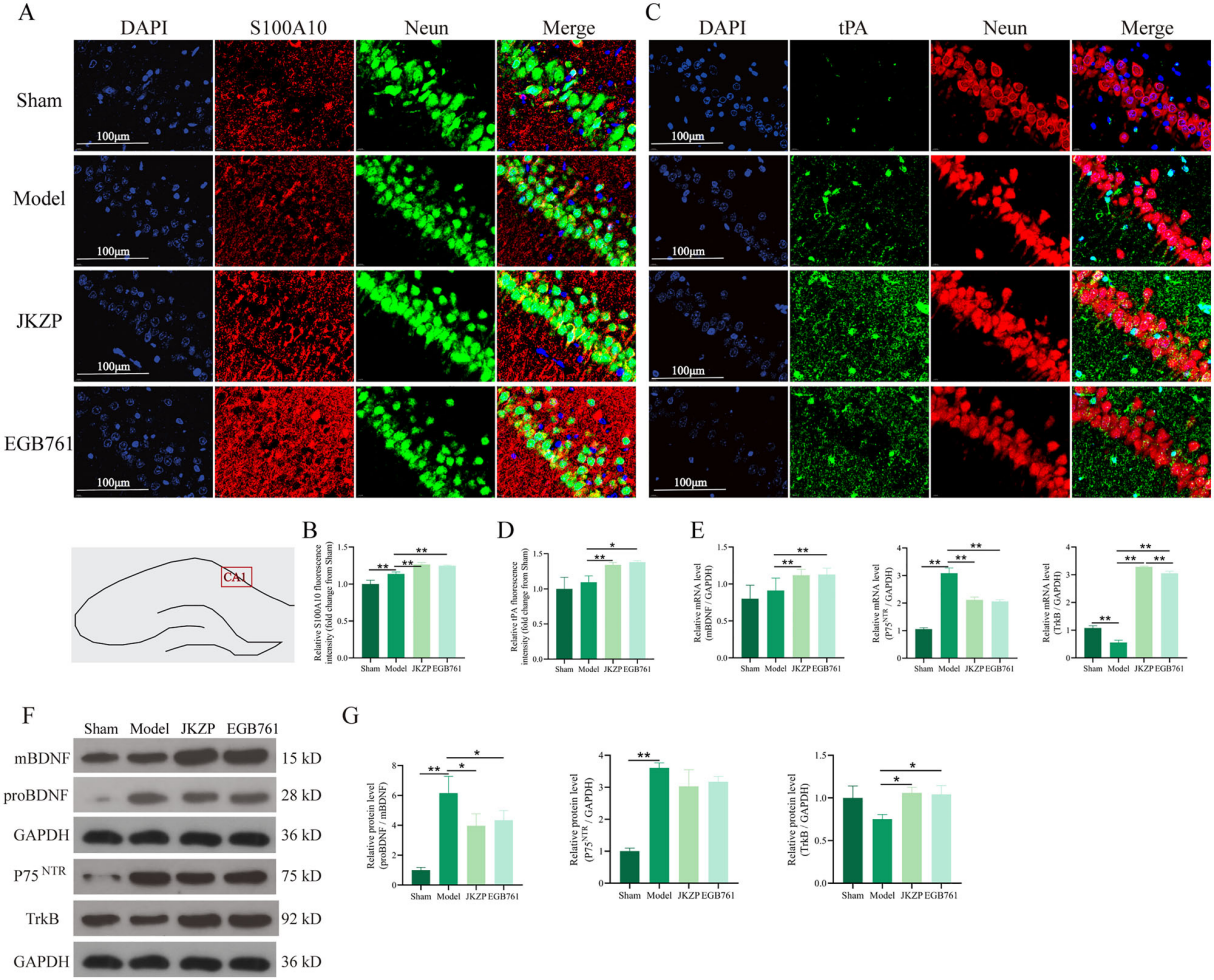
Effects of JKZP on S100A10/tPA/BDNF pathway. (A–D) Representative images of IF staining for S100A10 and tPA, and corresponding quantitative analyses (×1000); (E) RT‐qPCR analysis of detection of mBDNF, TrkB, and P75^NTR^ mRNA expression; (F and G) WB analysis of BDNF pathway protein expression. *p < 0.05, **p < 0.01. *n* = 3. JKZP, *Jiawei Kongsheng Zhenzhong Pill*; mBDNF, mature BDNF; P75NTR, P75 neurotrophic receptor; proBDNF, protein of BDNF; S100A10, S100 calcium‐binding protein A10; tPA, tissue‐type plasminogen activator; TrKB, tropomyosin receptor kinase B.

Studies have reported that neuronal synaptic plasticity is associated with the proBDNF/mBDNF balance and is regulated by S100A10/tPA (Ludka et al. 2017; Park et al. 2016; Wang et al. 2022; Yang et al. 2021). Therefore, the present study investigated the effect of JKZP on S100A10/tPA/BDNF pathway. As demonstrated in Figure 6A–D, S100A10 protein expression in the hippocampal CA1 region was increased in the model group compared with the sham group (*p *< 0.01), whereas tPA protein expression was increased but not statistically different (*p* > 0.05). Both S100A10 and tPA protein levels were significantly elevated in the JKZP and EGB761 groups compared with the model group (*p *< 0.05, *p *< 0.01). WB and RT‐qPCR assays for BDNF and downstream pathway‐related molecules revealed a significant increase in the proBDNF/mBDNF protein ratio in the model group when compared with the sham group (*p *< 0.01). mBDNF mRNA levels were elevated but not significantly (*p *> 0.05), P75^NTR^ mRNA level was increased (*p *< 0.01), and TrkB mRNA expression was decreased (*p *< 0.01). Compared with the model group, the ratio of proBDNF/mBDNF protein was decreased (*p *< 0.05), mBDNF and TrkB mRNA levels were increased in the JKZP and EGB761 groups (*p *< 0.01), whereas P75^NTR^ mRNA levels were significantly reduced (*p *< 0.01) (Figure 6E–G). It is suggested that JKZP may regulate proBDNF/mBDNF balance by modulating S100A10/tPA activity.

### 3.6 JKZP May Promote Survival of OGD/R‐Injured Primary Hippocampal Neurons by Regulating proBDNF/mBDNF Balance Through Upregulation of S100A10/tPA

**FIGURE 7:**
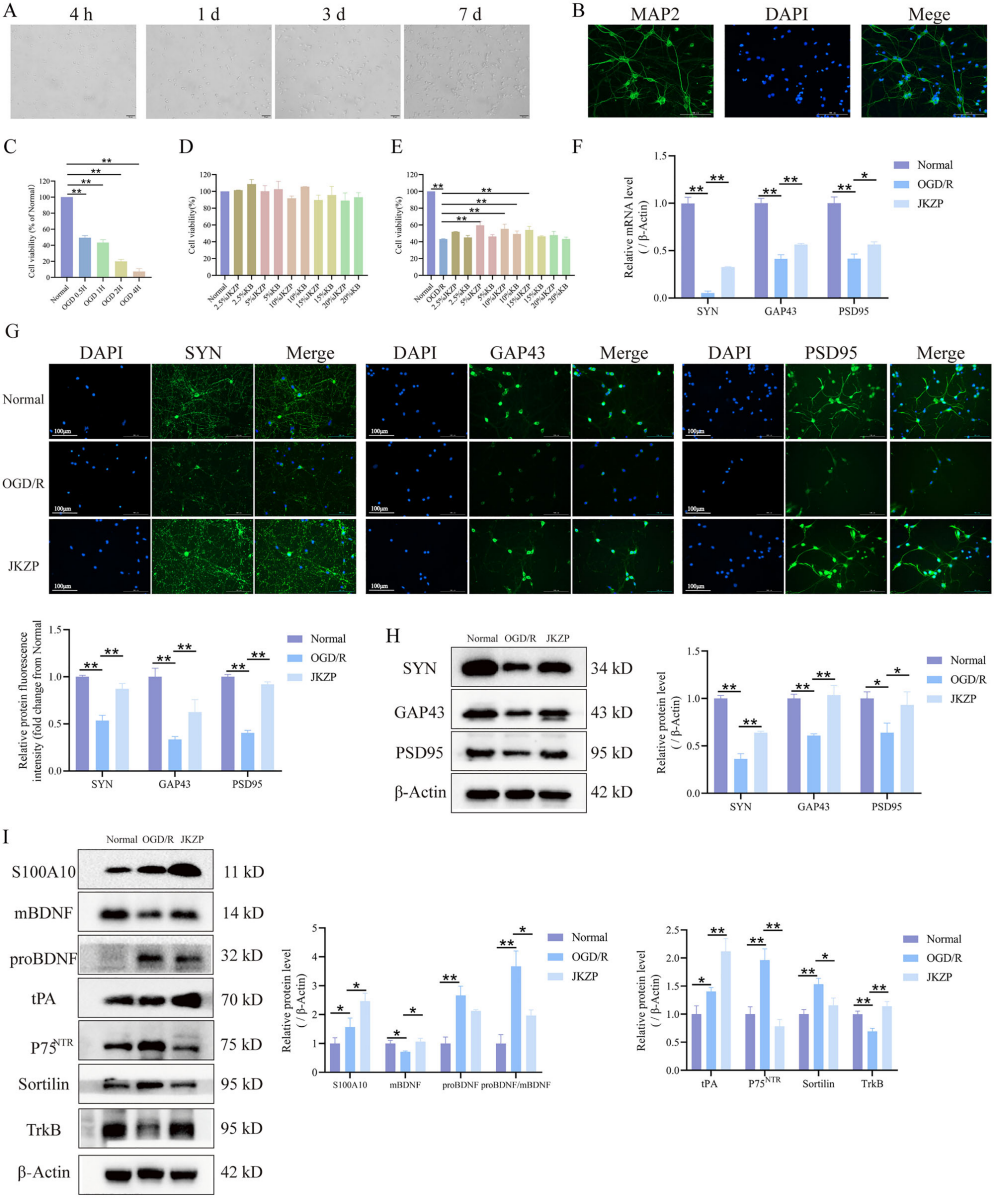
JKZP‐containing serum may enhance the synaptic plasticity of hippocampal neurons by interfering with S100A10/tPA and modulating the proBDNF/mBDNF balance. (A) Neuronal growth process (×200); (B) MAP2 immunofluorescence to identify the purity of neurons (the purity was >95%, ×200, *n* = 5); (C) CCK8 screening of neurons for OGD modeling time (*n* = 6); (D) CCK8 detection of the toxic effects of JKZP‐containing serum lyophilized powder on neurons (*n* = 5); (E) CCK8 detection of the effects of JKZP‐containing serum lyophilized powder on neuronal activity (*n* = 5). (F) RT‐qPCR assay for PSD95, GAP43, and SYN mRNA expressions (*n* = 3); (G) representative images of IF staining for SYN, GAP43, and PSD95 and corresponding quantitative analyses (*n* = 4, ×200); (H) WB assay for PSD95, GAP43, and SYN protein expressions (*n* = 3); (I) WB assay for S100A10/tPA/BDNF pathway related protein levels (*n* = 3). *p < 0.05, **p < 0.01. JKZP, *Jiawei Kongsheng Zhenzhong Pill*; mBDNF, mature BDNF; P75^NTR^, P75 neurotrophic receptor; proBDNF, protein of BDNF; S100A10, S100 calcium‐binding protein A10; tPA, tissue‐type plasminogen activator; TrKB, tropomyosin receptor kinase B.

Previous in vivo experiments indicated that JKZP may improve neuronal synaptic plasticity by modulating the S100A10/tPA/BDNF pathway, thereby exerting an anti‐MCI effect. To validate this role and mechanism in vitro, we successfully isolated and cultured primary rat hippocampal neurons (Figure 7A,B), constructed an in vitro OGD/R model (Figure 7C) and determined that a 5% JKZP‐containing serum lyophilized powder was the optimal condition for in vitro experiments (Figure 7D,E). Both SYN, GAP43, and PSD95 proteins and mRNA expression were significantly reduced in the OGD/R group compared with the normal group (*p* < 0.05, *p* < 0.01). However, JKZP treatment significantly reversed these reductions (*p* < 0.05, *p* < 0.01) (Figure 7F–H). These findings suggest that JKZP improves synaptic plasticity in hippocampal neurons in vitro.

Synaptic plasticity regulation in hippocampal neurons is closely related to S100A10/tPA. Compared with the normal group, S100A10, tPA, proBDNF/mBDNF, P75^NTR^, and sortilin protein levels were significantly higher in the OGD/R group (*p* < 0.05, *p* < 0.01), whereas the TrkB protein levels were significantly lower (*p* < 0.01). Compared with the OGD/R group, S100A10, tPA, and TrkB protein levels in the JKZP group were elevated (*p* < 0.05, *p* < 0.01), whereas proBDNF/mBDNF, P75^NTR^, and Sortilin protein levels were significantly decreased (*p* < 0.05, *p* < 0.01) (Figure 7I). It is suggested that the improvement in neuronal plasticity by JKZP may be related to the regulation of proBDNF/mBDNF balance by S100A10/tPA.

### 3.7 Silencing of S100A10 Abolished the Protective Effect of JKZP on Primary Hippocampal Neurons

**FIGURE 8:**
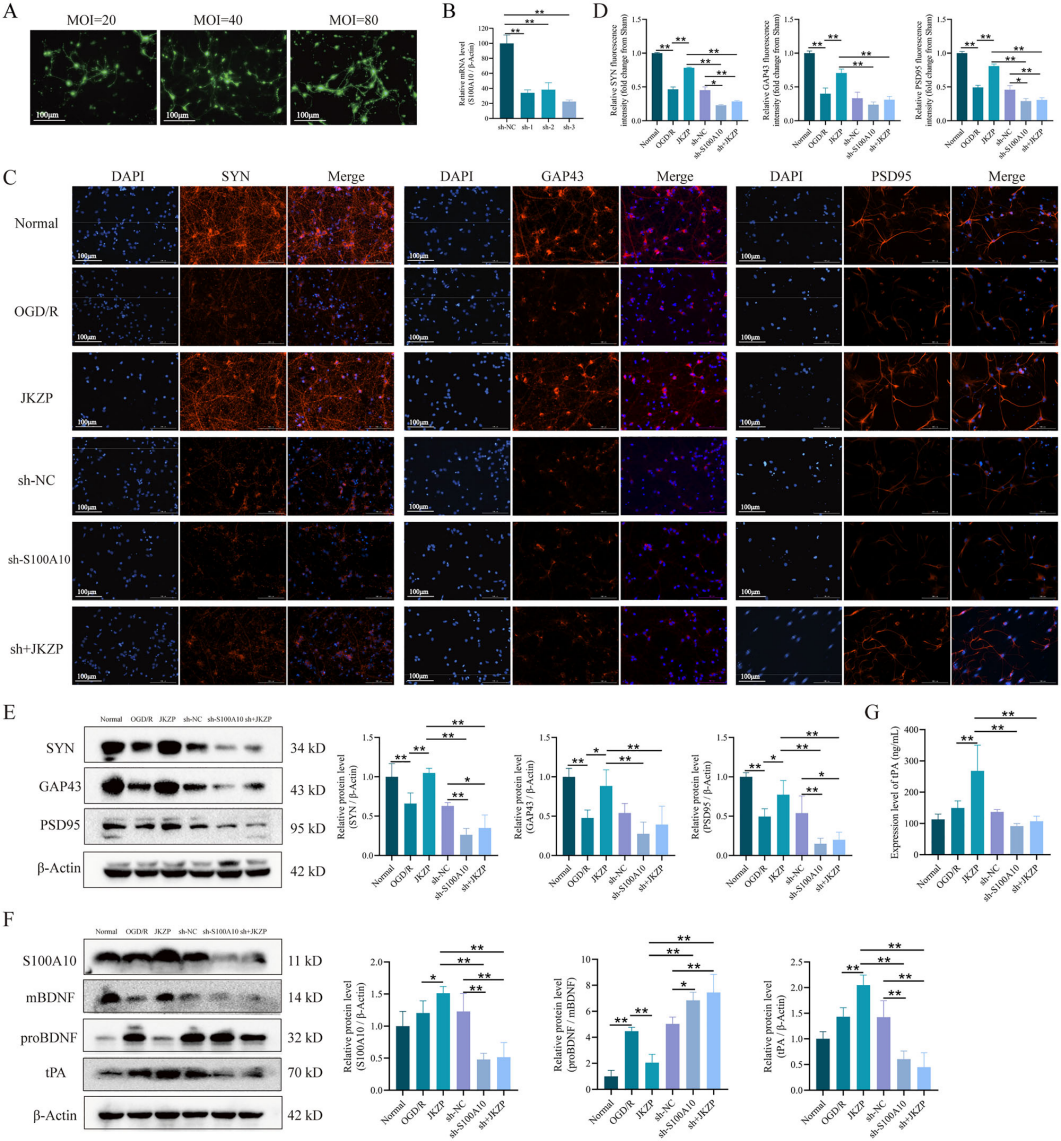
JKZP improves neuronal synaptic plasticity by regulating the balance of proBDNF/mBDNF through S100A10/tPA. (A) Fluorescence microscopy to observe the transfection rate (×200); (B) RT‐qPCR to detect the cellular S100A10 knockdown rate (*n* = 4); (C and D) representative images of IF staining for SYN, GAP43, and PSD95 and corresponding quantitative analyses (*n* = 4, ×200); (E) WB detection of synapse‐associated protein expression (*n* = 4); (F) WB assay for S100A10/tPA/BDNF pathway related protein expression (*n* = 4); (G) ELISA detection of tPA protein expression (*n* = 6). *p < 0.05, **p < 0.01. JKZP, *Jiawei Kongsheng Zhenzhong Pill*; mBDNF, mature BDNF; MOI, multiplicity of infection; OGD/R, oxygen‐glucose deprivation/reperfusion; proBDNF, protein of BDNF; S100A10, S100 calcium‐binding protein A10; tPA, tissue‐type plasminogen activator.

To explore the role of S100A10 in regulating neuronal synaptic plasticity, this study utilized RNAi technology to construct primary hippocampal neurons with S100A10 lentiviral knockdown. The experimental results indicated that a MOI of 80 was optimal, with sh‐3 being the selected fragment (Figure 8A,B). Compared with the normal group, the OGD/R group exhibited blurred axons, reduced cellular connections, and decreased protein expression levels of SYN, GAP43, and PSD95 (*p* < 0.05, *p* < 0.01), mildly elevated S100A10 and tPA (*p* > 0.05), and remarkably increased proBDNF/mBDNF ratios (*p* < 0.01). Compared with the OGD/R group, JKZP treatment significantly reversed the reductions of SYN, GAP43, and PSD95 expression (*p* < 0.05, *p* < 0.01), increased S100A10 and tPA expressions (*p* < 0.05, *p* < 0.01), alongside decreased proBDNF/mBDNF ratio (*p* < 0.01). However, the sh‐S100A10 group showed varying degrees of reduction in SYN, GAP43, PSD95, S100A10, and tPA levels and elevated proBDNF/mBDNF ratio (*p* < 0.05, *p* < 0.01), and JKZP failed to rescue the loss of synapse‐associated proteins and activate S100A10/tPA in the S100A10 knockdown neurons (Figure 8C–F). Meanwhile, tPA protein activity was assessed using ELISA, which revealed decreased tPA levels in the OGD/R and sh‐S100A10 groups compared with the normal group and increased tPA level in the JKZP group compared with the OGD/R group (*p* < 0.01) (Figure 8G). These findings suggest that JKZP targets S100A10/tPA to mediate the proBDNF/mBDNF balance to regulate the synaptic plasticity of hippocampal neurons, and silencing of S100A10 abolished the inhibitory effect of the protective effect of JKZP on neurons.

## 4 Discussion

With the aggravation of global population aging, MCI seriously impacts the quality of life for over one‐quarter of the elderly (aged ≥ 60 years) in rural communities in China (Cong et al. 2023). MCI is a precursor stage of VaD or other forms of dementia and is therefore a crucial target for prevention and treatment strategies of dementia. Guided by the theory of “superior treatment before sick” of TCM, early identification of MCI and timely interventions can effectively delay the onset of dementia. This represents a novel approach within TCM in preventing and treating dementia. Clinical trials and pharmacological studies have demonstrated that TCM is effective, safe, and reliable in MCI (Wang et al. 2019; Zhang et al. 2019). JKZP originates from the classic “Thousand‐Golden‐Prescriptions,” and is commonly used to treat dementia, amnesia, insomnia, and so forth. The constituents and their active ingredients in JKZP have been extensively studied and are known to promote neural stem cell proliferation and differentiation (Shu et al. 2014), enhance neuronal synaptic growth (Lyu and Jia 2022), and inhibit neuroinflammation (Deng et al. 2023). In this study, we identified 64 blood entry components of JKZP by Q‐Orbitrap‐HRMS technology, including phenolic acids, iridoid glycosides, phthalides, diterpene quinones, and other compounds. β‐Asarone originating from *A. tatarinowii* Schott and *L. sinense* “Chuanxiong,” ferulic acid from *L. sinense* “Chuanxiong” and *S. miltiorrhiza* Bunge, loganin from *C. officinalis* Sieb. et Zucc., senkyunolide H from *L. sinense* “Chuanxiong,” and cryptotanshinone from *S. miltiorrhiza* Bunge may be key contributors to the neuroprotective effects of this formula. It is important to note that the ability to cross the blood‐brain barrier (BBB) and target specific regions of the brain is an essential property of chemicals or components that appeared neuroprotective effects (Hornok et al. 2022; Katila et al. 2022). As previous studies have demonstrated, many of the components in JKZP can pass through the BBB (Li 2007; Wang et al. 2013; Yang 2019; Zhang et al. 2011; Zhu, Lu, and Li 2013).

EGB761 is a standardized *G. biloba* extract containing ginkgo flavonoid glycosides, terpene trilactone, and ginkgolides as active ingredients. It is recommended as the preferred choice of proprietary Chinese medicines for VaD in China with significant efficacy and well tolerance in patients with mild to moderate VaD (Group Standardization Project 2021). Numerous studies have confirmed that EGB761 treatment after CCH could improve spatial cognitive function by ameliorating synaptic plasticity impairment, synapse degeneration, and axon demyelination (Wang et al. 2006; Yao et al. 2021). In the current study, gavage administration of JKZP or EGB761 effectively enhanced learning, memory, and cognitive abilities in MCI rats. Histopathological analysis demonstrated that JKZP significantly ameliorated tissue deficits in the hippocampal CA1 area. These results suggest that JKZP is effective in treating MCI. The hippocampus, especially the CA1 area, is highly sensitive to ischemia and hypoxia (Kim et al. 2024) and is crucial for learning, spatial memory, and cognitive abilities, of which the neurons in the hippocampal CA1 area are especially important for memory functions (Cossart and Khazipov 2022). Cognitive impairment resulting from CCH is closely associated with alterations in hippocampal synaptic plasticity. MCI animal models exhibited abnormalities characterized by reduced dendritic spine density, abnormal expression of synapse‐related molecules such as SYN, GAP43, and PSD95, decreased vesicle density in the presynaptic membrane, sparse dense material in the postsynaptic membrane, and impaired long‐term potentiation (LTP) and long‐term depression (LTD) in the hippocampus (Lin et al. 2023; Yao et al. 2021). It is suggested that abnormal hippocampal synaptic structural plasticity following chronic cerebral ischemia may constitute the primary pathological changes in MCI and dementia. Promoting hippocampal synaptic repair and synaptic plasticity may be crucial in delaying MCI progression. TCM has been extensively studied in modulating synaptic plasticity to improve cognitive impairment (Deng et al. 2023; Kwon et al. 2020; Lyu and Jia 2022; Mahaman et al. 2023; Nie et al. 2021; Ningning et al. 2024). Our study also demonstrated that JKZP improved neuronal damage in the hippocampal CA1 region, increased dendritic spine density, and enhanced the mRNA and protein expression of synapse‐related molecules such as SYN, GAP43, and PSD95. These results favored the functional recovery of the damaged neurons, which was also verified in an in vitro OGD/R primary hippocampal neuron model. It suggested that JKZP can alleviate MCI symptoms and slow disease progression by enhancing neuronal synaptic plasticity to promote neuronal survival.

Synaptic plasticity is modulated by endogenous growth‐associated factors in brain tissues (Rossi, Gianola, and Corvetti 2007). BDNF and its receptor are widely expressed throughout the brain, especially in hippocampal tissues with the highest content. BDNF exists in two forms: mBDNF and proBDNF. mBDNF is positively correlated with neural activity and preferentially binds to the TrkB receptor to initiate intracellular signaling cascades that promote neuronal survival, enhance synaptic plasticity, and support neurogenesis to exert a neurorestorative effect. In contrast, proBDNF preferentially binds with its high‐affinity P75^NTR^/sortilin, promotes neuronal apoptosis, disrupts axonal growth cones through RhoA/Rock2 pathways, and mediates neurotoxic effects (Diniz et al. 2024; Hsu et al. 2024). Though proBDNF can be cleaved into mBDNF, under physiological conditions, it is often considered to be less active and potentially neurotoxic if not converted to the mature form. The balance between proBDNF and mBDNF is crucial in determining whether TrkB activation predominates (leading to neuroprotective effects) or whether the neurotoxic effects of proBDNF prevail. Generally, higher levels of mBDNF relative to proBDNF are associated with neuroprotective effects via TrkB signaling. Conversely, when the proBDNF/mBDNF ratio shifts toward proBDNF, it can promote neurotoxic effects via p75^NTR^ activation, especially under conditions where proBDNF cannot efficiently convert to mBDNF. However, no specific “threshold” has been universally defined across all contexts. Therefore, modulating proBDNF/mBDNF balance may present a novel approach to ameliorating synaptic plasticity damage and cognitive impairment. To investigate changes in the proBDNF/mBDNF balance in an MCI model, we carried out RT‐qPCR and WB techniques. The results revealed a significant elevation in proBDNF and P75^NTR^ levels, a significant decrease in TrkB levels, and an elevated proBDNF/mBDNF ratio in the hippocampal tissues of MCI rats. However, JKZP intervention significantly reversed the above phenomena. Meanwhile, similar changes were observed in the in vitro primary hippocampal neuron OGD/R model. These findings suggest that the enhancement of neuronal synaptic plasticity by JKZP is associated with the modulation of the proBDNF/mBDNF balance and activation of downstream signaling pathways.

JKZP promotes the conversion of hippocampal proBDNF to mBDNF in MCI rats, which may be the mechanism of JKZP against MCI. Regulating the proBDNF/mBDNF balance involves various proteases. BDNF is initially synthesized as proBDNF, which is either hydrolyzed intracellularly by furin and pro‐protein convertases hydrolyzed intracellularly or cleaved extracellularly by fibrinolytic enzymes and selective matrix metalloproteinases (MMPs) (e.g., MMP3, MMP7) to form mBDNF (Chao and Bothwell 2002; Lee et al. 2001; Yang et al. 2024). Therefore, regulating the related protease activities is crucial for modulating the proBDNF/mBDNF balance, which mediates neuronal synaptic activity. tPA is a key serine protease involved in the extracellular conversion of proBDNF to mBDNF. Elevated tPA expression in hippocampal neurons helps to activate the protease, facilitate the hydrolysis of the proBDNF to mBDNF conversion process, and improve neuronal synaptic plasticity (Yeh, Huang, and Hsu 2012), which is essential for promoting long‐term memory formation (Pang et al. 2004). It has also been reported that tPA not only functions as a fibrinolytic agent in the circulatory system to catalyze the conversion of fibrinogen to fibrinolytic enzymes and provides a favorable environment for axon regeneration but also promotes neuronal survival and axon regeneration in a manner that is not dependent on fibrinogen activation (Gan et al. 2021). S100A10, believed to be closely related to neurogenesis (Egeland et al. 2010), can modulate the neuroprotective effects of BDNF‐mediated neurotoxicity and improve synaptic plasticity (Park et al. 2016). Meanwhile, S100A10 is a crucial regulator of tPA activity initiation. Its carboxy‐terminal lysine binds to tPA to participate in the stimulation of tPA‐dependent activation of plasminogen to regulate the cleavage of proBDNF, which is essential for regulating the cleavage of proBDNF balance between proBDNF and mBDNF (Scaini et al. 2015). Both S100A10 and tPA have been implicated in neuroplasticity, injury response, and recovery. S100A10 could possibly affect tPA activity or expression indirectly, particularly in systems like the fibrinolytic process or neuronal signaling pathways, but they are not typically considered to have a direct receptor‐agonist relationship. Increased expression of S100A10 and tPA in hippocampal tissues was found to elevate mBDNF protein levels and the mBDNF/proBDNF ratio (Ludka et al. 2017), suggesting that improving S100A10/tPA‐mediated cleavage hydrolysis of proBDNF and regulating proBDNF/mBDNF homeostasis may be an important molecular target for improving neuroplasticity in MCI. Our results showed that the expression levels of S100A10 and tPA appeared elevated in the CA1 region of hippocampal tissues in MCI rats, and JKZP treatment significantly elevated the expression levels of both. Similar results were obtained in in vitro experiments. These findings indicate that the regulation of the hippocampal proBDNF/mBDNF homeostasis by JKZP may be related to the activation of S100A10/tPA. To further verify our hypothesis, we next successfully constructed S100A10 knockdown lentiviral primary hippocampal neurons with the help of RNAi technology. S100A10 knockdown neurons showed reduced expression levels of S100A10, tPA, and synapse‐associated proteins such as SYN, GAP43, and PSD95. Meanwhile, the proBDNF/mBDNF ratio was significantly elevated, and JKZP could not effectively reverse the loss of synapse‐associated proteins or the elevated proBDNF/mBDNF ratio in neurons with S100A10 knockdown. The results further demonstrate that JKZP mediates proBDNF/mBDNF balance by targeting S100A10/tPA to improve MCI neuroplasticity.

Various components contained in JKZP have been shown to exert anti‐cognitive effects by improving synaptic plasticity. *L. sinense* “Chuanxiong” extract and loganin can reverse the loss of neurons and synapses as well as the reduction of synaptic plasticity in the brain tissues of demented animals and improve their cognitive deficits (Deng et al. 2023; Nie et al. 2021). Cryptotanshinone promotes neuronal synapse growth and ameliorates synaptic damage by regulating the PI3K/Akt/GSK3β pathway (Lyu and Jia 2022). Ferulic acid can effectively increase PSD95 and SYN synaptic protein expression in dementia models to improve synaptic plasticity and cognitive dysfunction (Mahaman et al. 2023). These findings help to further support our conclusion that JKZP can treat MCI by targeting S100A10/tPA to modulate proBDNF/mBDNF balance. Currently, our study has limitations, such as the lack of in vivo inverse experimental validation. Additionally, we did not further clarify the specific components of JKZP targeting regulation of S100A10/tPA and did not carry out the cerebrospinal fluid pharmacology and brain tissue metabolic distribution study of JKZP, which are also the focus of our next study to further improve the pharmacological substance base study and the site of action of the neuroprotective effect exerted by JKZP.

## 5 Conclusion

In this study, we investigated the role and mechanism of JKZP in treating an MCI model. Our findings showed that JKZP improved hippocampal neuroplasticity, alleviated MCI symptoms, and slowed dementia progression by targeting S100A10/tPA‐mediated modulation of proBDNF/mBDNF homeostasis. Notably, these findings provide a new theoretical basis and possible target for delaying MCI progression by TCM and provide new insights into the interpretation of the biological mechanism of tonifying the kidney and generating medulla oblongata to promote the beneficial effect. At the same time, the study provides a reliable basis for the clinical treatment and application of JKZP in the treatment of cognitive impairment disorders.

## Author Contributions

**Qiaolan Wu**: writing–original draft, methodology, formal analysis, data curation. **Yang Zhou and Chunxue Ou**: methodology, data curation. **Zu Gao, Xiaolin Wu, Yue Zhao, and Yuan Wang**: visualization, validation, investigation, data curation. **Zhichun Wu and Huayun Yu**: writing–review and editing, supervision, resources, project administration, methodology, funding acquisition, conceptualization.

## Ethics Statement

All experiments adhered to China's Regulations on the Management of Laboratory Animals, and the study animal experiments were approved by the Animal Ethics Committee of the Shandong University of TCM, with an ethical approval number of SDUTCM20221021004.

## Conflicts of Interest

The authors declare no conflicts of interest.

### Peer Review

The peer review history for this article is available at https://publons.com/publon/10.1002/brb3.70328.

## Supporting information



Supporting Information

## Data Availability

The data used to support the findings of this study are included in the article and are available upon reasonable request.
