# Supplementary material for: Jiawei Kongsheng Zhenzhong Pill (JKZP) Alleviates Chronic Cerebral Hypoperfusion‐Induced Hippocampal Synaptic Damage via S100A10/tPA/BDNF Pathway
Source: Brain Behav. 2025 Feb 17;15(2):e70328. doi: 10.1002/brb3.70328 (PMC11830996; doi:10.1002/brb3.70328)
Supplement: Supplementary file 1 — Supporting Information [file BRB3-15-e70328-s001.docx]

**Supplementary materials**


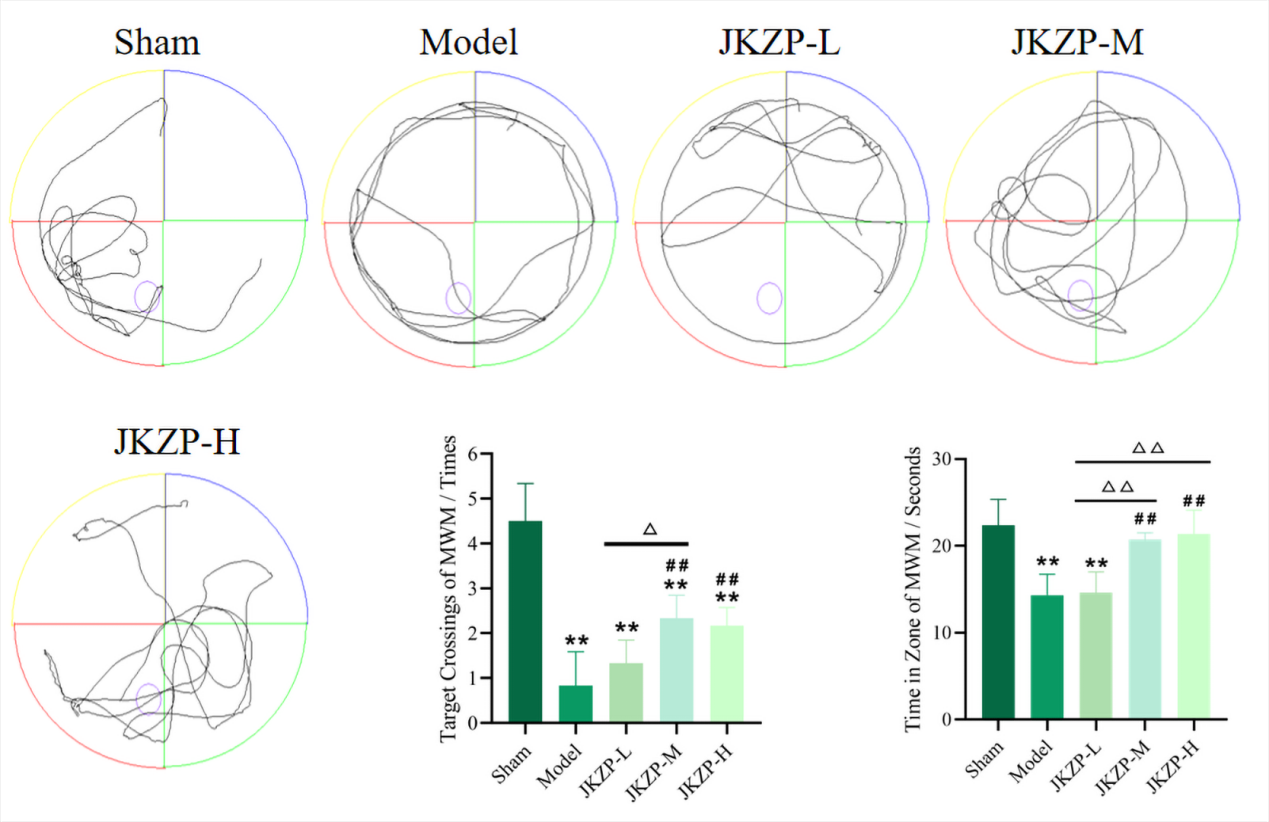


**supplementary Figure S1.** MWM detected the influence of JKZP on the learning and memory ability of MCI rats (n=6). vs Sham, **P* < 0.05, ***P* < 0.01; vs Model, ^#^*P* < 0.05, ^# #^*P* < 0.01; vs JKZP-L, ^△^*P* < 0.05, ^△△^*P* < 0.01.

**Supplementary Table 1**

Components of JKZP aqueous extract

| **No.** | **Name** | **Formula** | **Molecular Weight** | **RT [min]** | **mzCloud Best Match** |
| --- | --- | --- | --- | --- | --- |
| 1 | 1,2-Dipalmitoylphosphatidylglycerol | C38 H75 O10 P | 744.49148 | 25.047 | 97.7 |
| 2 | Ursolic acid | C30 H48 O3 | 456.35915 | 16.78 | 95.6 |
| 3 | L-Norleucine | C6 H13 N O2 | 131.09443 | 1.395 | 95.6 |
| 4 | L-Phenylalanine | C9 H11 N O2 | 165.07865 | 2.469 | 95.5 |
| 5 | Choline | C5 H13 N O | 103.09988 | 0.951 | 95.2 |
| 6 | Bis(4-ethylbenzylidene)sorbitol | C24 H30 O6 | 414.2027 | 12.821 | 93.8 |
| 7 | Adenosine | C10 H13 N5 O4 | 267.09588 | 2.317 | 93.7 |
| 8 | β-Asarone | C12 H16 O3 | 208.10936 | 12.056 | 93.5 |
| 9 | Oleamide | C18 H35 N O | 264.24449 | 17.544 | 93.1 |
| 10 | Citroflex A-4 | C20 H34 O8 | 402.22394 | 16.157 | 93 |
| 11 | Betaine | C5 H11 N O2 | 117.07892 | 1.034 | 92.9 |
| 12 | Loganin | C17 H26 O10 | 390.15091 | 6.184 | 92.8 |
| 13 | 5-Hydroxymethyl-2-furaldehyde | C6 H6 O3 | 126.03154 | 3.14 | 92.8 |
| 14 | Senkyunolide H | C12 H16 O4 | 206.09315 | 8.58 | 92.4 |
| 15 | Cryptotanshinone | C19 H20 O3 | 296.13995 | 14.143 | 92.3 |
| 16 | 2,4,5-Trimethoxybenzaldehyde | C10 H12 O4 | 196.07267 | 8.738 | 92.3 |
| 17 | Citroflex 2 | C12 H20 O7 | 276.11989 | 9.815 | 92.2 |
| 18 | Erucamide | C22 H43 N O | 320.30628 | 20.738 | 92 |
| 19 | 2,3,4,9-Tetrahydro-1H-β-carboline-3-carboxylic acid | C12 H12 N2 O2 | 216.08895 | 5.428 | 91.5 |
| 20 | DL-Stachydrine | C7 H13 N O2 | 143.0943 | 0.988 | 91.4 |
| 21 | Arctiin | C27 H34 O11 | 551.2345 | 8.961 | 91.4 |
| 22 | Tanshinone IIA | C19 H18 O3 | 294.12463 | 15.296 | 91.2 |
| 23 | Phloretin | C15 H14 O5 | 274.08308 | 8.337 | 91.1 |
| 24 | PEG n5 | C10 H22 O6 | 238.14138 | 4.86 | 91.1 |
| 25 | L-Tyrosine | C9 H11 N O3 | 181.07345 | 1.316 | 91.1 |
| 26 | N1-Acetylspermine | C12 H28 N4 O | 244.22598 | 3.91 | 91 |
| 27 | 3,4-Dimethyl-2,5-bis(3,4,5-trimethoxyphenyl)tetrahydrofuran | C24 H32 O7 | 432.21341 | 13.605 | 91 |
| 28 | 4-Dodecylbenzenesulfonic acid | C18 H30 O3 S | 326.19063 | 23.789 | 90.9 |
| 29 | Indole-3-acrylic acid | C11 H9 N O2 | 187.063 | 4.214 | 90 |
| 30 | DL-Carnitine | C7 H15 N O3 | 161.10469 | 0.927 | 90 |
| 31 | Sedanolide | C12 H18 O2 | 194.13027 | 13.053 | 89.9 |
| 32 | 5'-S-Methyl-5'-thioadenosine | C11 H15 N5 O3 S | 297.08963 | 5.154 | 89.8 |
| 33 | N-Butylbenzenesulfonamide | C10 H15 N O2 S | 213.0818 | 0.127 | 89.7 |
| 34 | Palmitoyl ethanolamide | C18 H37 N O2 | 299.28142 | 16.582 | 89.6 |
| 35 | Guanine | C5 H5 N5 O | 134.02258 | 1.976 | 89.6 |
| 36 | PEG n6 | C12 H26 O7 | 282.16777 | 5.275 | 89.5 |
| 37 | 3-Hydroxy-2-methylpyridine | C6 H7 N O | 109.05295 | 1.274 | 89.3 |
| 38 | Nicotinic acid | C6 H5 N O2 | 123.03203 | 1.291 | 89 |
| 39 | (6,6-Dimethylbicyclo[3.1.1]hept-2-yl)methyl 6-O-[(2R,3R,4R)-3,4-dihydroxy-4-(hydroxymethyl)tetrahydro-2-furanyl]-β-D-glucopyranoside | C21 H36 O10 | 465.25567 | 8.986 | 89 |
| 40 | Urocanic acid | C6 H6 N2 O2 | 138.04258 | 1.009 | 88.9 |
| 41 | 2,2'-Methylenebis(4-methyl-6-tert-butylphenol) | C23 H32 O2 | 340.23884 | 17.203 | 88.9 |
| 42 | Salvianolic acid B | C36 H30 O16 | 735.17548 | 8.529 | 88.6 |
| 43 | Adenine | C5 H5 N5 | 135.05423 | 1.269 | 88.6 |
| 44 | 3-Hydroxypicolinic acid | C6 H5 N O3 | 139.02671 | 1.321 | 88.6 |
| 45 | Zerumbone | C15 H22 O | 218.16635 | 11.456 | 88.5 |
| 46 | D-(+)-Camphor | C10 H16 O | 152.11929 | 7.906 | 88.5 |
| 47 | Stearamide | C18 H37 N O | 283.28635 | 18.963 | 88.4 |
| 48 | Taurochenodeoxycholic acid | C26 H45 N O6 S | 499.29509 | 12.972 | 88.3 |
| 49 | Caffeic acid | C9 H8 O4 | 180.04099 | 8.051 | 88.3 |
| 50 | Ageratriol | C15 H24 O3 | 234.16113 | 11.583 | 88.3 |
| 51 | Nootkatone | C15 H22 O | 218.16635 | 11.932 | 88.2 |
| 52 | DL-Arginine | C6 H14 N4 O2 | 157.08473 | 1.054 | 88.2 |
| 53 | Phenethylamine | C8 H11 N | 104.06268 | 3.173 | 88 |
| 54 | 10-HDA | C10 H18 O3 | 168.11424 | 7.887 | 87.8 |
| 55 | 9S,13R-12-Oxophytodienoic acid | C18 H28 O3 | 292.20297 | 10.091 | 87.7 |
| 56 | Berberine | C20 H17 N O4 | 335.11358 | 8.203 | 87.5 |
| 57 | N,N'-Dicyclohexylurea | C13 H24 N2 O | 224.18805 | 11.304 | 87.4 |
| 58 | Hexadecanamide | C16 H33 N O | 255.25528 | 17.288 | 87.4 |
| 59 | PEG n10 | C20 H42 O11 | 458.27096 | 6.729 | 87.3 |
| 60 | Sorbic acid | C6 H8 O2 | 112.05263 | 4.522 | 87.2 |
| 61 | N-Feruloyloctopamine | C18 H19 N O5 | 311.11453 | 7.505 | 87 |
| 62 | Schisandrin | C24 H32 O7 | 432.2134 | 11.849 | 86.9 |
| 63 | Genistein | C15 H10 O5 | 270.05234 | 12.929 | 86.9 |
| 64 | 1-Linoleoyl glycerol | C21 H38 O4 | 354.27565 | 16.757 | 86.9 |
| 65 | Nicotinamide | C6 H6 N2 O | 122.048 | 1.568 | 86.5 |
| 66 | 4-Indolecarbaldehyde | C9 H7 N O | 145.05228 | 7.851 | 86.5 |
| 67 | 2-Isopropylmalic acid | C7 H12 O5 | 176.06716 | 5.042 | 86.4 |
| 68 | Palmitoyl sphingomyelin | C39 H79 N2 O6 P | 702.56503 | 27.402 | 86.3 |
| 69 | 3,5-di-tert-Butyl-4-hydroxybenzaldehyde | C15 H22 O2 | 234.16122 | 7.273 | 86.3 |
| 70 | Proline | C5 H9 N O2 | 115.06348 | 1.056 | 86.2 |
| 71 | PEG n12 | C24 H50 O13 | 546.32337 | 7.188 | 86.2 |
| 72 | PEG n11 | C22 H46 O12 | 502.29694 | 6.984 | 86 |
| 73 | Stearic acid | C18 H36 O2 | 284.27066 | 20.003 | 85.9 |
| 74 | Octyl decyl phthalate | C26 H42 O4 | 418.3068 | 21.018 | 85.9 |
| 75 | 6-Hydroxy-5a,9-dimethyl-3-methylene-3a,4,5,5a,6,7,9a,9b-octahydronaphtho[1,2-b]furan-2(3H)-one | C15 H20 O3 | 248.14033 | 11.066 | 85.8 |
| 76 | Oleanolic acid | C30 H48 O3 | 438.34843 | 12.52 | 85.7 |
| 77 | 3-Indoxyl sulphate | C8 H7 N O4 S | 213.00833 | 6.543 | 85.5 |
| 78 | 3-Methoxycinnamic acid | C10 H10 O3 | 160.05184 | 11.51 | 85.1 |
| 79 | (5ξ)-12,13-Dihydroxypodocarpa-8,11,13-trien-7-one | C17 H22 O3 | 274.15628 | 12.086 | 85.1 |
| 80 | Avocadyne 1-acetate | C19 H34 O4 | 308.23404 | 17.207 | 85 |
| 81 | Salvianolic acid A | C26 H22 O10 | 494.11896 | 7.528 | 84.8 |
| 82 | Tridemorph | C19 H39 N O | 297.3022 | 19.292 | 84.7 |
| 83 | cis,cis-Muconic acid | C6 H6 O4 | 142.02656 | 4.279 | 84.6 |
| 84 | 3-Methylhistidine | C7 H11 N3 O2 | 169.08478 | 0.867 | 84.5 |
| 85 | Hippuric acid | C9 H9 N O3 | 179.0568 | 5.419 | 84.4 |
| 86 | 2'-O-Methyladenosine | C11 H15 N5 O4 | 281.11202 | 3.908 | 84.2 |
| 87 | Glycyl-L-leucine | C8 H16 N2 O3 | 188.11584 | 2.51 | 84.1 |
| 88 | Arachidonic acid | C20 H32 O2 | 304.23885 | 17.366 | 84.1 |
| 89 | 2,4-Dimethylbenzaldehyde | C9 H10 O | 134.073 | 4.981 | 84 |
| 90 | Dodecamethylcyclohexasiloxane | C12 H36 O6 Si6 | 444.11149 | 16.803 | 83.9 |
| 91 | 4-Ethylbenzaldehyde | C9 H10 O | 134.07281 | 10.195 | 83.9 |
| 92 | Asiatic acid | C30 H48 O5 | 488.34883 | 12.298 | 83.7 |
| 93 | PEG n16 | C32 H66 O17 | 739.45365 | 7.791 | 83.6 |
| 94 | Ferulic acid | C10 H10 O4 | 194.05691 | 7.318 | 83.6 |
| 95 | cis-5,8,11,14,17-Eicosapentaenoic acid | C20 H30 O2 | 302.22367 | 16.575 | 83.6 |
| 96 | Oleoyl ethanolamide | C20 H39 N O2 | 307.28648 | 17.945 | 83.5 |
| 97 | D-(+)-Maltose | C12 H22 O11 | 364.0969 | 1.01 | 83.5 |
| 98 | Caprolactam | C6 H11 N O | 113.0844 | 4.944 | 83.3 |
| 99 | D-Raffinose | C18 H32 O16 | 526.14964 | 0.944 | 83.2 |
| 100 | 1-Stearoylglycerol | C21 H42 O4 | 358.30694 | 19.047 | 83.2 |
| 101 | 7-Methyl-3-methylene-6-(3-oxobutyl)-3,3a,4,7,8,8a-hexahydro-2H-cyclohepta[b]furan-2-one | C15 H20 O3 | 230.12982 | 13.067 | 83 |
| 102 | (3aS,10aR,10bR)-6,10a-Dimethyl-3-methylene-3,3a,4,5,7,8,10a,10b-octahydrofuro[3',2':6,7]cyclohepta[1,2-b]pyran-2,9-dione | C15 H18 O4 | 262.11937 | 9.583 | 83 |
| 103 | (±)-Abscisic acid | C15 H20 O4 | 264.13515 | 9.026 | 82.9 |
| 104 | Bis(2-ethylhexyl) amine | C16 H35 N | 241.27626 | 12.967 | 82.8 |
| 105 | 3',4'-Dihydroxyphenylacetone | C9 H10 O3 | 166.06239 | 5.453 | 82.8 |
| 106 | Methyl indole-3-acetate | C11 H11 N O2 | 189.07832 | 9.263 | 82.7 |
| 107 | 4,5-Dicaffeoylquinic acid | C25 H24 O12 | 516.1254 | 7.742 | 82.7 |
| 108 | (-)-Caryophyllene oxide | C15 H24 O | 220.18183 | 10.847 | 82.7 |
| 109 | 3-(4-Methoxyphenyl)-5-[(4-nitrophenoxy)methyl]-4,5-dihydroisoxazole | C17 H16 N2 O5 | 350.09082 | 12.839 | 82.4 |
| 110 | 3-Hydroxydecanoic acid | C10 H20 O3 | 188.13989 | 11.943 | 82.3 |
| 111 | Suberic acid | C8 H14 O4 | 174.08789 | 7.123 | 82.2 |
| 112 | **Pentadecanoic acid** | C15 H30 O2 | 242.22359 | 17.678 | 82.2 |
| 113 | BMK methyl glycidate | C11 H12 O3 | 192.07812 | 6.642 | 82.2 |
| 114 | Azelaic acid | C9 H16 O4 | 188.10348 | 8.232 | 82.2 |
| 115 | Diphenylamine | C12 H11 N | 169.08872 | 13.211 | 82 |
| 116 | Neochlorogenic acid | C16 H18 O9 | 354.09401 | 5.49 | 81.8 |
| 117 | Cyclopiazonic acid | C20 H20 N2 O3 | 336.14626 | 13.062 | 81.8 |
| 118 | (9aR,9bS)-9a-Hydroxy-6,9-dimethyl-3-methylene-3,3a,4,5,9a,9b-hexahydroazuleno[4,5-b]furan-2,7-dione | C15 H16 O4 | 260.10393 | 9.765 | 81.7 |
| 119 | Sinapinic acid | C11 H12 O5 | 206.05674 | 7.904 | 81.5 |
| 120 | (3S,4R)-3-(1-hydroxyhexyl)-4-(hydroxymethyl)oxolan-2-one | C11 H20 O4 | 198.12492 | 11.129 | 81.3 |
| 121 | Dibutyl phthalate | C16 H22 O4 | 278.15102 | 14.032 | 81.2 |
| 122 | Butyl benzoate | C11 H14 O2 | 178.09862 | 8.714 | 81.2 |
| 123 | 3-Methoxybenzaldehyde | C8 H8 O2 | 136.05202 | 12.202 | 81.2 |
| 124 | Docosanamide | C22 H45 N O | 339.34887 | 20.586 | 81.1 |
| 125 | 4-Hydroxymandelic acid | C8 H8 O4 | 186.05129 | 5.773 | 81.1 |
| 126 | DL-Lysine | C6 H14 N2 O2 | 146.10526 | 0.803 | 81 |
| 127 | 7,8-Bis(hydroxymethyl)-1,4a-dimethyl-3,4,4a,5,6,7-hexahydro-2H-benzo[7]annulen-2-one | C15 H22 O3 | 250.15593 | 10.205 | 81 |
| 128 | 4-[4-(4-Hydroxy-3-methoxyphenyl)tetrahydro-1H,3H-furo[3,4-c]furan-1-yl]-2-methoxyphenyl hexopyranoside | C26 H32 O11 | 566.19873 | 7.768 | 81 |
| 129 | (8aR,12S,12aR)-12-Hydroxy-4-methyl-4,5,6,7,8,8a,12,12a-octahydro-2H-3-benzoxecine-2,9(1H)-dione | C14 H20 O4 | 252.13528 | 9.766 | 81 |
| 130 | PEG n13 | C26 H54 O14 | 590.34903 | 7.387 | 80.9 |
| 131 | Cannabichromevarin | C19 H26 O2 | 286.19243 | 12.807 | 80.9 |
| 132 | (2E)-3-(4-Hydroxyphenyl)-N-[2-(4-hydroxyphenyl)ethyl]acrylamide | C17 H17 N O3 | 283.11948 | 8.621 | 80.7 |
| 133 | Chlorogenic acid | C16 H18 O9 | 354.09395 | 5.284 | 80.5 |
| 134 | 4-Ethoxybenzaldehyde | C9 H10 O2 | 150.06757 | 6.185 | 80.5 |
| 135 | Mussaenosidic acid | C16 H24 O10 | 376.13574 | 5.061 | 80.4 |
| 136 | Maltotetraose | C24 H42 O21 | 688.2016 | 0.956 | 80.3 |
| 137 | DL-Tryptophan | C11 H12 N2 O2 | 204.08867 | 4.22 | 80.3 |
| 138 | 2-Amino-4-methylpyrimidine | C5 H7 N3 | 109.06411 | 22.525 | 80.2 |
| 139 | (7R,8S)-7,8-Dihydroxy-3,7-dimethyl-6-oxo-7,8-dihydro-6H-isochromene-5-carbaldehyde | C12 H12 O5 | 236.0678 | 7.634 | 80.1 |
| 140 | Cyclo(phenylalanyl-prolyl) | C14 H16 N2 O2 | 244.12045 | 7.472 | 80 |
| 141 | Vanillin | C8 H8 O3 | 152.04686 | 3.622 | 79.9 |
| 142 | Eucalyptol | C10 H18 O | 136.12496 | 7.798 | 79.8 |
| 143 | (3R,4S)-4,6,8-Trihydroxy-7-methoxy-3-methyl-3,4-dihydro-1H-isochromen-1-one | C11 H12 O6 | 222.05199 | 7.385 | 79.6 |
| 144 | α-Estradiol | C18 H24 O2 | 272.177 | 13.604 | 79.5 |
| 145 | trans-Cinnamaldehyde | C9 H8 O | 132.05714 | 6.186 | 79.5 |
| 146 | Sibiricose A1 | C23 H32 O15 | 548.17262 | 5.778 | 79.5 |
| 147 | 6-Methylquinoline | C10 H9 N | 143.07297 | 5.426 | 79.4 |
| 148 | (3aR,8R,8aR,9aR)-8-Hydroxy-8a-methyl-3,5-bis(methylene)decahydronaphtho[2,3-b]furan-2(3H)-one | C15 H20 O3 | 248.14034 | 9.464 | 79.4 |
| 149 | 4-Ethoxy ethylbenzoate | C11 H14 O3 | 194.09377 | 12.869 | 79.1 |
| 150 | PPG n8 | C24 H50 O9 | 499.37014 | 10.779 | 79 |
| 151 | 2-Hydroxypropazine | C9 H17 N5 O | 211.14314 | 6.623 | 79 |
| 152 | 2-Anisic acid | C8 H8 O3 | 134.03627 | 9.499 | 79 |
| 153 | Uridine | C9 H12 N2 O6 | 244.06877 | 1.439 | 78.9 |
| 154 | 6α-Prostaglandin I1 | C20 H34 O5 | 336.22928 | 13.051 | 78.9 |
| 155 | (15Z)-9,12,13-Trihydroxy-15-octadecenoic acid | C18 H34 O5 | 330.23962 | 10.51 | 78.9 |
| 156 | 16-Hydroxyhexadecanoic acid | C16 H32 O3 | 272.23452 | 14.155 | 78.8 |
| 157 | Myristyl sulfate | C14 H30 O4 S | 294.18583 | 23.102 | 78.7 |
| 158 | Leucylproline | C11 H20 N2 O3 | 228.14695 | 4.483 | 78.6 |
| 159 | Irgafos 168 | C42 H63 O3 P | 646.44939 | 24.796 | 78.6 |
| 160 | Cantharidin | C10 H12 O4 | 196.07267 | 6.33 | 78.6 |
| 161 | Docosahexaenoic acid | C22 H32 O2 | 328.23901 | 17.126 | 78.4 |
| 162 | Halcinonide | C24 H32 Cl F O5 | 454.1951 | 13.645 | 78.2 |
| 163 | Ambrosic acid | C15 H20 O4 | 246.1245 | 9.027 | 78.2 |
| 164 | 6:2 Fluorinated telomer sulfonate | C8 H5 F13 O3 S | 427.97373 | 15.361 | 78.2 |
| 165 | Dodecyl sulfate | C12 H26 O4 S | 266.15437 | 20.981 | 78 |
| 166 | PEG n14 | C28 H58 O15 | 634.37454 | 7.554 | 77.9 |
| 167 | Corchorifatty acid F | C18 H32 O5 | 328.22412 | 10.068 | 77.8 |
| 168 | 3,4-Dihydroxyphenylpropionic acid | C9 H10 O4 | 164.04715 | 6.737 | 77.6 |
| 169 | 3-Hydroxybenzyl alcohol | C7 H8 O2 | 124.0508 | 5.773 | 77.5 |
| 170 | 3,4-Dihydroxybenzenesulfonic acid | C6 H6 O5 S | 189.99235 | 2.83 | 77.5 |
| 171 | 2-(3,4-Dihydroxyphenyl)ethyl 3-O-(6-deoxy-β-L-mannopyranosyl)-6-O-[(2E)-3-(3,4-dihydroxyphenyl)-2-propenoyl]-β-D-glucopyranoside | C29 H36 O15 | 624.20333 | 7.711 | 77.5 |
| 172 | 1,3-Benzodioxolylbutanamine (BDB) | C11 H15 N O2 | 193.10981 | 27.287 | 77.4 |
| 173 | Triethyl phosphate | C6 H15 O4 P | 182.07035 | 8.341 | 77.3 |
| 174 | Isoferulic acid | C10 H10 O4 | 194.05718 | 5.474 | 77.3 |
| 175 | Ethyl paraben | C9 H10 O3 | 166.06242 | 5.223 | 77.3 |
| 176 | Ethyl levulinate | C7 H12 O3 | 144.0763 | 5.427 | 77.3 |
| 177 | Tolycaine | C15 H22 N2 O3 | 278.16238 | 6.517 | 77.2 |
| 178 | Syringic acid | C9 H10 O5 | 198.05138 | 6.025 | 77.2 |
| 179 | 3,4-Dihydroxybenzaldehyde | C7 H6 O3 | 138.03119 | 5.451 | 77.2 |
| 180 | PPG n5 | C15 H32 O6 | 308.21893 | 8.417 | 77.1 |
| 181 | Isophthalic acid | C8 H6 O4 | 166.02509 | 5.502 | 77.1 |
| 182 | Isocytosine | C4 H5 N3 O | 111.04338 | 1.054 | 77 |
| 183 | D-Glucosamine | C6 H13 N O5 | 179.07907 | 1.211 | 77 |
| 184 | Linoleic acid | C18 H32 O2 | 280.23904 | 17.581 | 76.9 |
| 185 | Oleic acid | C18 H34 O2 | 282.25475 | 18.689 | 76.8 |
| 186 | Sucrose | C12 H22 O11 | 342.11497 | 1.032 | 76.6 |
| 187 | 9-Oxo-10(E),12(E)-octadecadienoic acid | C18 H30 O3 | 294.21829 | 10.501 | 76.6 |
| 188 | Tretinoin | C20 H28 O2 | 300.20801 | 15.641 | 76.5 |
| 189 | 5-[2-(3-Furyl)ethyl]-8a-(hydroxymethyl)-5,6-dimethyl-3,4,4a,5,6,7,8,8a-octahydro-1-naphthalenecarboxylic acid | C20 H28 O4 | 332.19776 | 10.705 | 76.5 |
| 190 | 3,5-di-tert-Butylbenzaldehyde | C15 H22 O | 218.16636 | 19.481 | 76.5 |
| 191 | Phlinoside A | C35 H46 O20 | 786.25588 | 6.571 | 76.4 |
| 192 | 3-Phenyllactic acid | C9 H10 O3 | 166.0616 | 6.87 | 76.4 |
| 193 | 4'-Methoxyacetophenone | C9 H10 O2 | 150.06764 | 9.697 | 76.3 |
| 194 | 2,3-Dihydroxybenzoic acid | C7 H6 O4 | 154.02517 | 3.781 | 76.2 |
| 195 | (3R,5R)-1,3,5-Trihydroxy-4-{[(2E)-3-(4-hydroxy-3-methoxyphenyl)-2-propenoyl]oxy}cyclohexanecarboxylic acid | C17 H20 O9 | 368.10972 | 6.61 | 76.2 |
| 196 | Paracetamol | C8 H9 N O2 | 151.06299 | 7.125 | 76.1 |
| 197 | 2,5-Dihydroxybenzaldehyde | C7 H6 O3 | 138.03007 | 4.943 | 76 |
| 198 | Bioside | C20 H30 O12 | 462.17271 | 4.849 | 75.9 |
| 199 | α-Eleostearic acid | C18 H30 O2 | 278.22377 | 16.738 | 75.8 |
| 200 | Cyclo(leucylprolyl) | C11 H18 N2 O2 | 210.13656 | 6.871 | 75.8 |
| 201 | 3-Coumaric acid | C9 H8 O3 | 164.04599 | 6.884 | 75.8 |
| 202 | Δ2-trans-Hexadecenoic acid | C16 H30 O2 | 254.22366 | 17.22 | 75.7 |
| 203 | Citric acid | C6 H8 O7 | 192.02566 | 1.326 | 75.7 |
| 204 | 3-Hydroxybenzoic acid | C7 H6 O3 | 138.03008 | 7.678 | 75.5 |
| 205 | Rutin | C27 H30 O16 | 632.14065 | 19.848 | 75.4 |
| 206 | Gibberellin A7 | C19 H22 O5 | 348.15635 | 10.175 | 75.4 |
| 207 | Apocynin | C9 H10 O3 | 166.06244 | 10.746 | 75.4 |
| 208 | Ethyl myristate | C16 H32 O2 | 256.23921 | 18.436 | 75.3 |
| 209 | Shogaol | C17 H24 O3 | 276.17163 | 11.773 | 75 |
| 210 | 5-Methoxysalicylic acid | C8 H8 O4 | 168.04217 | 5.745 | 74.8 |
| 211 | Indole-3-acetic acid | C10 H9 N O2 | 175.0625 | 7.202 | 74.7 |
| 212 | Geniposidic acid | C16 H22 O10 | 374.12037 | 4.388 | 74.7 |
| 213 | Butyl 4-aminobenzoate | C11 H15 N O2 | 193.10966 | 8.916 | 74.7 |
| 214 | Uric acid | C5 H4 N4 O3 | 168.02679 | 1.022 | 74.6 |
| 215 | Decanamide | C10 H21 N O | 171.16182 | 12.231 | 74.5 |
| 216 | Pyridoxal | C8 H9 N O3 | 167.05795 | 1.552 | 74.3 |
| 217 | Methyl palmitate | C17 H34 O2 | 287.28152 | 11.12 | 74.2 |
| 218 | Zearalenone | C18 H22 O5 | 300.13506 | 11.686 | 74.1 |
| 219 | Myristic acid | C14 H28 O2 | 228.20785 | 16.952 | 74.1 |
| 220 | β-Estradiol | C18 H24 O2 | 272.17684 | 13.257 | 74 |
| 221 | 4-Methylumbelliferyl-α-D-glucopyranoside | C16 H18 O8 | 338.09965 | 5.657 | 74 |
| 222 | 4-Anisic acid | C8 H8 O3 | 152.04592 | 6.693 | 74 |
| 223 | Methyl (1S,7R)-1-(β-D-glucopyranosyloxy)-7-methyl-5-oxo-1,4a,5,6,7,7a-hexahydrocyclopenta[c]pyran-4-carboxylate | C17 H24 O10 | 388.13521 | 5.451 | 73.9 |
| 224 | 4-(4-Ethoxyphenyl)-4-oxobutanoic acid | C12 H14 O4 | 222.08809 | 10.034 | 73.8 |
| 225 | 2-NMC | C12 H17 N O | 191.13036 | 9.814 | 73.7 |
| 226 | 6-Hydroxy-8-methoxy-3-methyl-3,4-dihydro-1H-isochromen-1-one | C11 H12 O4 | 208.07271 | 5.454 | 73.5 |
| 227 | Mevalonolactone | C6 H10 O3 | 130.06289 | 1.993 | 73.4 |
| 228 | Glycerophospho-N-palmitoyl ethanolamine | C21 H44 N O7 P | 453.28388 | 14.856 | 73.4 |
| 229 | 2-Hydroxycinnamic acid | C9 H8 O3 | 146.03596 | 9.539 | 73.3 |
| 230 | 1,2,3,4-Tetramethyl-1,3-cyclopentadiene | C9 H14 | 122.10925 | 7.877 | 73.2 |
| 231 | (2R)-5-Methoxy-2-methyl-2,3,8,9-tetrahydro-4H-furo[2,3-H]chromen-4-one | C13 H14 O4 | 234.08841 | 9.498 | 73.2 |
| 232 | Methyl cinnamate | C10 H10 O2 | 162.0675 | 7.947 | 73.1 |
| 233 | 2-(1-Adamantyl)-5H-thieno[3',2':5,6]thiino[4,3-d]pyrimidine | C19 H20 N2 S2 | 340.1063 | 11.217 | 73 |
| 234 | 2-Furoic acid | C5 H4 O3 | 112.01626 | 1.787 | 72.7 |
| 235 | 7-(3,4-Dihydroxyphenyl)-1-(4-hydroxyphenyl)-3-heptanyl acetate | C21 H26 O5 | 380.15863 | 13.094 | 72.5 |
| 236 | 3-tert-Butyladipic acid | C10 H18 O4 | 202.11932 | 9.173 | 72.5 |
| 237 | Methyl 1-(hexopyranosyloxy)-4a-hydroxy-7-methyl-5-oxo-1,4a,5,6,7,7a-hexahydrocyclopenta[c]pyran-4-carboxylate | C17 H24 O11 | 450.1364 | 5.312 | 72.4 |
| 238 | 1,6-Bis-O-(3,4,5-trihydroxybenzoyl)hexopyranose | C20 H20 O14 | 484.08418 | 5.371 | 72.4 |
| 239 | 5-Phenylcyclohexane-1,3-dione | C12 H12 O2 | 188.08297 | 9.777 | 72.3 |
| 240 | 1-(β-D-Glucopyranosyloxy)-7-methyl-1,4a,5,6,7,7a-hexahydrocyclopenta[c]pyran-4-carboxylic acid | C16 H24 O9 | 360.14133 | 7.129 | 72.3 |
| 241 | 1,5-Anhydro-2-O-(6-O-benzoyl-α-L-galactopyranosyl)-D-glucitol | C19 H26 O11 | 476.15179 | 5.901 | 72.2 |
| 242 | 18-β-Glycyrrhetinic acid | C30 H46 O4 | 470.33805 | 12.706 | 72.1 |
| 243 | 3,3'-Diisopropyl-6,6'-dimethyl-2,2',5,5'-biphenyltetrol | C20 H26 O4 | 330.18226 | 11.839 | 72 |
| 244 | 2-Mercaptobenzothiazole | C7 H5 N S2 | 166.98488 | 10.72 | 72 |
| 245 | Resorcinol monoacetate | C8 H8 O3 | 152.04594 | 5.684 | 71.8 |
| 246 | Citrinin | C13 H14 O5 | 250.08345 | 9.712 | 71.7 |
| 247 | Shanzhiside methyl ester | C17 H26 O11 | 452.15233 | 4.968 | 71.6 |
| 248 | 2-[(2S,4aR,8aS)-2-Hydroxy-4a-methyl-8-methylenedecahydro-2-naphthalenyl]acrylic acid | C15 H22 O3 | 250.15609 | 15.951 | 71.6 |
| 249 | Gluconic acid | C6 H12 O7 | 196.05689 | 1.029 | 71.5 |
| 250 | (3β,5ξ,9ξ)-3,23-Dihydroxy-1-oxoolean-12-en-28-oic acid | C30 H46 O5 | 486.33327 | 12.171 | 71.3 |
| 251 | PEG n15 | C30 H62 O16 | 695.42741 | 7.699 | 71.2 |
| 252 | D-(-)-Quinic acid | C7 H12 O6 | 192.06199 | 1.018 | 71.2 |
| 253 | Fenpropimorph | C20 H33 N O | 303.25523 | 16.259 | 71.1 |
| 254 | 2-Methylbenzoic acid | C8 H8 O2 | 136.05081 | 8.237 | 71.1 |
| 255 | Creatine | C4 H9 N3 O2 | 131.06932 | 1.035 | 70.9 |
| 256 | 2-Hydroxyestradiol | C18 H24 O3 | 288.17165 | 11.647 | 70.9 |
| 257 | Cathinone | C9 H11 N O | 149.08381 | 1.273 | 70.7 |
| 258 | N-Isovalerylglycine | C7 H13 N O3 | 159.08795 | 6.206 | 70.6 |
| 259 | Lauric acid | C12 H24 O2 | 200.17632 | 15.465 | 70.6 |
| 260 | 5-Hydroxyindole-3-acetic acid | C10 H9 N O3 | 191.0578 | 0.13 | 70.5 |
| 261 | Indole-3-lactic acid | C11 H11 N O3 | 205.07376 | 4.09 | 70.4 |
| 262 | 3-Hydroxymandelic acid | C8 H8 O4 | 168.04073 | 3.369 | 70.4 |
| 263 | 6-Quinolinecarboxylic acid | C10 H7 N O2 | 173.04741 | 0.49 | 70.2 |
| 264 | Kynurenic acid | C10 H7 N O3 | 189.0419 | 4.177 | 70.1 |
| 265 | D-(+)-Glucose | C6 H12 O6 | 197.08966 | 1.23 | 70.1 |
| 266 | 2-Hydroxy-4,5',8a'-trimethyl-1'-oxo-4-vinyloctahydro-1'H-spiro[cyclopentane-1,2'-naphthalene]-5'-carboxylic acid | C20 H30 O4 | 334.21347 | 10.203 | 70.1 |
| 267 | 10-Nitrolinoleate | C18 H31 N O4 | 307.21304 | 8.82 | 70.1 |
| 268 | 6-Phenoxynicotinic acid | C12 H9 N O3 | 215.05747 | 6.97 | 69.9 |
| 269 | {(1R,2R)-2-[(2Z)-5-(Hexopyranosyloxy)-2-penten-1-yl]-3-oxocyclopentyl}acetic acid | C18 H28 O9 | 388.17222 | 6.183 | 69.9 |
| 270 | Columbianetin | C14 H14 O4 | 246.08826 | 10.893 | 69.8 |
| 271 | 3-(2,6-Dihydroxyphenyl)-4-hydroxy-6-methyl-2-benzofuran-1(3H)-one | C15 H12 O5 | 272.06785 | 8.178 | 69.8 |
| 272 | Scoparone | C11 H10 O4 | 206.0569 | 8.396 | 69.7 |
| 273 | 4-Hydroxybenzaldehyde | C7 H6 O2 | 122.03515 | 5.506 | 69.7 |
| 274 | 2-Methoxyestrone | C19 H24 O3 | 300.17157 | 13.669 | 69.6 |
| 275 | Magnolol | C18 H18 O2 | 266.1298 | 12.603 | 69.4 |
| 276 | N-Acetyl-L-phenylalanine | C11 H13 N O3 | 207.08866 | 7.125 | 69.3 |
| 277 | L-(+)-Tartaric acid | C4 H6 O6 | 150.01486 | 1.069 | 69 |
| 278 | Dimethyl sebacate | C12 H22 O4 | 230.15117 | 13.057 | 69 |
| 279 | 1-[2-(1,3-Benzodioxol-5-yl)-3-methyl-1-benzofuran-5-yl]-1,2-propanediol | C19 H18 O5 | 308.10355 | 12.349 | 69 |
| 280 | Hydrocinnamic acid | C9 H10 O2 | 150.06654 | 7.576 | 68.7 |
| 281 | 2-Hydroxyhippuric acid | C9 H9 N O4 | 217.03545 | 0.9 | 68.7 |
| 282 | 1,2,3,4-Tetrahydroisoquinoline-1-acetic acid | C11 H13 N O2 | 191.09446 | 4.112 | 68.7 |
| 283 | Promethazine sulfoxide | C17 H20 N2 O S | 278.15094 | 15.144 | 68.5 |
| 284 | γ-Linolenic acid ethyl ester | C20 H34 O2 | 312.26526 | 17.489 | 68.3 |
| 285 | L-Pyroglutamic acid | C5 H7 N O3 | 129.04258 | 1.309 | 68.3 |
| 286 | Flurandrenolide | C24 H33 F O6 | 436.22667 | 6.431 | 68.3 |
| 287 | Benzoic acid | C7 H6 O2 | 122.03517 | 6.016 | 68.3 |
| 288 | 7-Hydroxycoumarine | C9 H6 O3 | 162.0311 | 6.559 | 68.2 |
| 289 | (3R,4S)-6,8-Dihydroxy-3,4,5-trimethyl-1-oxo-3,4-dihydro-1H-isochromene-7-carboxylic acid | C13 H14 O6 | 266.07886 | 5.899 | 68.2 |
| 290 | Dihomo-γ-linolenic acid ethyl ester | C22 H38 O2 | 340.29653 | 19.046 | 67.8 |
| 291 | Prostaglandin F2α 1-11-lactone | C20 H32 O4 | 318.21857 | 15.361 | 67.7 |
| 292 | Perillartine | C10 H15 N O | 165.11546 | 5.138 | 67.7 |
| 293 | (+/-)-C75 | C14 H22 O4 | 236.14025 | 9.573 | 67.7 |
| 294 | Matairesinol | C20 H22 O6 | 358.14013 | 10.497 | 67.4 |
| 295 | Formononetin | C16 H12 O4 | 268.07214 | 8.811 | 67.4 |
| 296 | Cetrimonium | C19 H41 N | 283.32305 | 14.09 | 67.4 |
| 297 | Pyrogallol | C6 H6 O3 | 126.03009 | 1.963 | 67.3 |
| 298 | Kanosamine | C6 H13 N O5 | 161.0685 | 0.808 | 67.3 |
| 299 | 4-Methoxybenzaldehyde | C8 H8 O2 | 136.05136 | 3.966 | 67.3 |
| 300 | α-Linolenoyl ethanolamide | C20 H35 N O2 | 321.26555 | 16.518 | 67.1 |
| 301 | Adenosine 3'5'-cyclic monophosphate | C10 H12 N5 O6 P | 329.05035 | 1.105 | 67.1 |
| 302 | 3-(4-Methylbenzoyl)acrylic acid | C11 H10 O3 | 190.06222 | 10.241 | 67.1 |
| 303 | DEET | C12 H17 N O | 191.1308 | 4.592 | 67 |
| 304 | Alternariolmethylether | C15 H12 O5 | 272.06751 | 9.529 | 67 |
| 305 | Picolinic acid | C6 H5 N O2 | 123.03038 | 1.312 | 66.9 |
| 306 | Phenacetin | C10 H13 N O2 | 179.09428 | 6.794 | 66.9 |
| 307 | 3-(1-hydroxyethyl)-2,3,6,7,8,8a-hexahydropyrrolo[1,2-a]pyrazine-1,4-dione | C9 H14 N2 O3 | 198.10002 | 3.157 | 66.9 |
| 308 | Piceatannol | C14 H12 O4 | 244.07253 | 10.751 | 66.8 |
| 309 | Lariciresinol 4-O-glucoside | C26 H34 O11 | 539.23419 | 7.894 | 66.6 |
| 310 | 7,8-Dihydroxy-4-methylcoumarin | C10 H8 O4 | 192.04123 | 6.657 | 66.4 |
| 311 | Nalorphine | C19 H21 N O3 | 311.15114 | 12.251 | 66.3 |
| 312 | N-(2,4-Dimethylphenyl)formamide | C9 H11 N O | 149.08379 | 1.434 | 66.3 |
| 313 | Diosmetin | C16 H12 O6 | 300.06243 | 11.77 | 66.3 |
| 314 | 5(Z),8(Z),11(Z)-Eicosatrienoic acid ethanolamide | C22 H39 N O2 | 349.29679 | 17.37 | 66.3 |
| 315 | Valpromide | C8 H17 N O | 143.13064 | 10.086 | 66.2 |
| 316 | N-Cyclohexyl-N-methyl-5-(2-pyridinyl)-2-thiophenesulfonamide | C16 H20 N2 O2 S2 | 314.11421 | 13.225 | 66 |
| 317 | (+/-)12(13)-DiHOME | C18 H34 O4 | 314.24505 | 12.959 | 65.8 |
| 318 | 4-Oxoproline | C5 H7 N O3 | 129.04093 | 1.044 | 65.7 |
| 319 | 2,2,6,6-Tetramethyl-4-piperidinol | C9 H19 N O | 157.14622 | 11.195 | 65.7 |
| 320 | Hexadecanedioic acid | C16 H30 O4 | 286.21372 | 10.766 | 65.6 |
| 321 | Gallic acid | C7 H6 O5 | 170.02004 | 1.884 | 65.5 |
| 322 | 6,18,19-Trihydroxytrachyloban-2-one | C20 H30 O4 | 334.21328 | 12.35 | 65.5 |
| 323 | 4-Phenolsulfonic acid | C6 H6 O4 S | 173.99723 | 5.754 | 65.5 |
| 324 | 2-Hydroxyphenylalanine | C9 H11 N O3 | 181.0735 | 6.108 | 65.5 |
| 325 | (+)-ar-Turmerone | C15 H20 O | 216.15073 | 13.194 | 65.4 |
| 326 | α-Lactose | C12 H22 O11 | 388.12043 | 1 | 65.2 |
| 327 | 2-Hydroxy-4-(4-hydroxyphenyl)butanoic acid | C10 H12 O4 | 196.0724 | 7.268 | 65.2 |
| 328 | Acetanilide | C8 H9 N O | 135.06814 | 1.087 | 65.1 |
| 329 | (5R,6S)-5-Hydroxy-4-methoxy-6-(2-phenylethyl)-5,6-dihydro-2H-pyran-2-one | C14 H16 O4 | 248.10403 | 7.4 | 65.1 |
| 330 | Normorphine | C16 H17 N O3 | 271.12018 | 6.316 | 65 |
| 331 | Coumarin | C9 H6 O2 | 146.03596 | 9.06 | 65 |
| 332 | 2-Methoxyresorcinol | C7 H8 O3 | 140.04657 | 4.642 | 64.9 |
| 333 | 16α-Hydroxyestrone | C18 H22 O3 | 286.15598 | 14.87 | 64.9 |
| 334 | β-Lapachone | C15 H14 O3 | 242.09345 | 11.357 | 64.8 |
| 335 | Jasmonic acid | C12 H18 O3 | 210.12432 | 11.278 | 64.6 |
| 336 | N-Acetyl-DL-glutamic acid | C7 H11 N O5 | 189.06234 | 1.026 | 64.4 |
| 337 | 3-Hydroxy-3,5,5-trimethyl-4-(3-oxo-1-buten-1-ylidene)cyclohexyl β-D-glucopyranoside | C19 H30 O8 | 408.17421 | 6.13 | 64.3 |
| 338 | Methanandamide | C23 H39 N O2 | 361.29661 | 18.051 | 64.2 |
| 339 | (2E)-3-[4-({2-O-[(2S,3R,4R)-3,4-Dihydroxy-4-(hydroxymethyl)tetrahydro-2-furanyl]-β-D-glucopyranosyl}oxy)-3-methoxyphenyl]acrylic acid | C21 H28 O13 | 488.15183 | 5.162 | 64.2 |
| 340 | Isotretinoin | C20 H28 O2 | 300.20805 | 14.317 | 64.1 |
| 341 | 4-(4-Ethoxyphenyl)-4-oxobut-2-enoic acid | C12 H12 O4 | 220.07247 | 9.676 | 64.1 |
| 342 | Gibberellin A4 | C19 H24 O5 | 332.16112 | 11.749 | 63.9 |
| 343 | Aflatoxin G2 | C17 H14 O7 | 330.07295 | 8.005 | 63.9 |
| 344 | Cytidine | C9 H13 N3 O5 | 243.08498 | 1.07 | 63.8 |
| 345 | 8-Iso-15-keto-prostaglandin-F2β | C20 H32 O5 | 334.2136 | 12.848 | 63.7 |
| 346 | 4-Methoxycinnamic acid | C10 H10 O3 | 178.06233 | 6.188 | 63.5 |
| 347 | 1,2,4-Benzenetricarboxylic acid | C9 H6 O6 | 210.01543 | 5.142 | 63.4 |
| 348 | 2-(6-Hydroxyhexyl)-3-methylenesuccinic acid | C11 H18 O5 | 230.11438 | 7.391 | 63.3 |
| 349 | Trendione | C18 H20 O2 | 268.14555 | 15.603 | 63.1 |
| 350 | Norharman | C11 H8 N2 | 168.06853 | 5.797 | 63.1 |
| 351 | 4-(2,7-Dihydroxy-6-methyl-2-heptanyl)-3-hydroxybenzoic acid | C15 H22 O5 | 282.14605 | 10.647 | 63.1 |
| 352 | 1-Naphthyl acetic acid | C12 H10 O2 | 186.06678 | 9.433 | 63.1 |
| 353 | U-50488 | C19 H26 Cl2 N2 O | 368.1433 | 7.599 | 63 |
| 354 | Citraconic acid | C5 H6 O4 | 130.02492 | 2.148 | 63 |
| 355 | D-Mannitol 1-phosphate | C6 H15 O9 P | 262.0471 | 7.666 | 62.9 |
| 356 | 4-Hydroxy-3-[2-(2-hydroxyphenyl)-2-oxoethyl]-2H-chromen-2-one | C17 H12 O5 | 296.06688 | 8.813 | 62.7 |
| 357 | 5-Hydroxytryptophan | C11 H12 N2 O3 | 220.0843 | 4.204 | 62.4 |
| 358 | Sorbicillin | C14 H16 O3 | 232.10893 | 12.196 | 62.3 |
| 359 | 4-Methoxycinnamaldehyde | C10 H10 O2 | 162.06749 | 14.66 | 62.3 |
| 360 | Kinetin | C10 H9 N5 O | 215.08045 | 4.61 | 62.1 |
| 361 | 2-(Methylsulfonyl)-3-(pyrazin-2-ylamino)acrylonitrile | C8 H8 N4 O2 S | 224.03463 | 18.274 | 62.1 |
| 362 | Tetranor-12(S)-HETE | C16 H26 O3 | 288.17161 | 11.088 | 62 |
| 363 | Bavachinin | C21 H22 O4 | 338.14815 | 14.192 | 62 |
| 364 | 2-Phenoxypropanoic acid | C9 H10 O3 | 166.06157 | 7.1 | 62 |
| 365 | L-(-)-Malic acid | C4 H6 O5 | 134.01979 | 1 | 61.8 |
| 366 | Butylparaben | C11 H14 O3 | 194.09319 | 9.936 | 61.8 |
| 367 | Oxohongdenafil | C25 H32 N6 O4 | 480.25276 | 6.736 | 61.7 |
| 368 | 4-Methylumbelliferone | C10 H8 O3 | 176.04673 | 8.932 | 61.5 |
| 369 | Sibiricose A3 | C19 H26 O13 | 462.13645 | 5.021 | 61.3 |
| 370 | Oleyl anilide | C24 H39 N O | 363.31232 | 17.808 | 61.3 |
| 371 | Desethylatrazine | C6 H10 Cl N5 | 187.06316 | 7.135 | 61.3 |
| 372 | 3-[2-(3-Hydroxyphenyl)ethyl]-5-methoxyphenol | C15 H16 O3 | 244.10911 | 11.674 | 61.3 |
| 373 | Phenylacetylglycine | C10 H11 N O3 | 193.07393 | 1.556 | 60.7 |
| 374 | 4-tert-Butylcyclohexyl acetate | C12 H22 O2 | 198.1615 | 15.961 | 60.6 |
| 375 | 15-Deoxy-δ12,14 -Prostaglandin J2 | C20 H28 O3 | 316.20289 | 14.189 | 60.6 |
| 376 | 4-Phenylbutyric acid | C10 H12 O2 | 164.08088 | 0.833 | 60.5 |
| 377 | 4-Cyclopentylphenol | C11 H14 O | 162.10288 | 10.768 | 60.5 |
| 378 | Fusarenon-X | C17 H22 O8 | 336.11951 | 11.215 | 60.4 |
| 379 | Coenzyme Q2 | C19 H26 O4 | 318.18214 | 12.134 | 60.3 |
| 380 | Asparagine | C4 H8 N2 O3 | 132.05332 | 1.098 | 60.2 |
| 381 | 3-Methoxysalicylic acid | C8 H8 O4 | 168.04411 | 7.648 | 60.2 |
| 382 | Eicosapentaenoic acid | C20 H30 O2 | 308.23424 | 14.238 | 60.1 |
| 383 | 1,5-Bis(2,5-dimethoxyphenyl)pentane-1,5-dione | C21 H24 O6 | 372.15573 | 8.959 | 60.1 |

**Supplementary Table 2**

Components in the JKZP-containing serum

| **No.** | **Name** | **Formula** | **Molecular Weight** | **RT [min]** | **mzCloud Best Match** |
| --- | --- | --- | --- | --- | --- |
| 1 | 1,2-Dipalmitoylphosphatidylglycerol | C38 H75 O10 P | 744.49148 | 25.047 | 97.7 |
| 2 | Senkyunolide H | C12 H16 O4 | 206.09315 | 8.58 | 92.4 |
| 3 | Cryptotanshinone | C19 H20 O3 | 296.13995 | 14.143 | 92.3 |
| 4 | 9-Oxo-10(E),12(E)-octadecadienoic acid | C18 H30 O3 | 294.21873 | 14.921 | 92.3 |
| 5 | Loganin | C17 H26 O10 | 407.17848 | 6.21 | 92.1 |
| 6 | 2,3,4,9-Tetrahydro-1H-β-carboline-3-carboxylic acid | C12 H12 N2 O2 | 216.08895 | 5.428 | 91.5 |
| 7 | PEG n5 | C10 H22 O6 | 238.14138 | 4.86 | 91.1 |
| 8 | PEG n10 | C20 H42 O11 | 458.27096 | 6.729 | 87.3 |
| 9 | Sorbic acid | C6 H8 O2 | 112.05263 | 4.522 | 87.2 |
| 10 | 3-Methoxycinnamic acid | C10 H10 O3 | 160.05185 | 9.628 | 86.8 |
| 11 | 3,5-di-tert-Butyl-4-hydroxybenzaldehyde | C15 H22 O2 | 234.16122 | 7.273 | 86.3 |
| 12 | PEG n12 | C24 H50 O13 | 546.32337 | 7.188 | 86.2 |
| 13 | PEG n11 | C22 H46 O12 | 502.29694 | 6.984 | 86 |
| 14 | PPG n9 | C27 H56 O10 | 540.38537 | 11.451 | 83.7 |
| 15 | Asiatic acid | C30 H48 O5 | 488.34883 | 12.298 | 83.7 |
| 16 | PEG n16 | C32 H66 O17 | 739.45365 | 7.791 | 83.6 |
| 17 | Ferulic acid | C10 H10 O4 | 194.05691 | 7.318 | 83.6 |
| 18 | 16α-Hydroxyestrone | C18 H22 O3 | 286.15632 | 12.089 | 83.6 |
| 19 | PPG n7 | C21 H44 O8 | 424.30216 | 10.072 | 83.5 |
| 20 | Taurocholic acid | C26 H45 N O7 S | 515.29051 | 9.812 | 82.6 |
| 21 | PPG n11 | C33 H68 O12 | 656.46858 | 12.673 | 82.5 |
| 22 | 4-Methoxycinnamic acid | C10 H10 O3 | 178.06228 | 9.629 | 82.3 |
| 23 | PPG n10 | C30 H62 O11 | 598.4272 | 12.074 | 81.7 |
| 24 | 8-Iso-15-keto-prostaglandin-F2β | C20 H32 O5 | 334.21365 | 13.701 | 81.7 |
| 25 | PEG n13 | C26 H54 O14 | 590.34903 | 7.387 | 80.9 |
| 26 | 4-Hydroxymandelic acid | C8 H8 O4 | 168.04072 | 5.754 | 80.8 |
| 27 | PEG n15 | C30 H62 O16 | 678.40164 | 7.688 | 80.1 |
| 28 | PEG n14 | C28 H58 O15 | 651.40184 | 7.452 | 80.1 |
| 29 | PPG n8 | C24 H50 O9 | 482.34382 | 10.79 | 79.6 |
| 30 | α-Estradiol | C18 H24 O2 | 272.177 | 13.604 | 79.5 |
| 31 | 6-Methylquinoline | C10 H9 N | 143.07297 | 5.426 | 79.4 |
| 32 | 5-Hydroxyindole | C8 H7 N O | 133.05259 | 6.594 | 79.1 |
| 33 | Phenylacetylglycine | C10 H11 N O3 | 193.07326 | 5.747 | 79 |
| 34 | Flurandrenolide | C24 H33 F O6 | 414.24523 | 1.055 | 78.8 |
| 35 | 6:2 Fluorinated telomer sulfonate | C8 H5 F13 O3 S | 427.97373 | 15.361 | 78.2 |
| 36 | PPG n5 | C15 H32 O6 | 308.21893 | 8.417 | 77.1 |
| 37 | Kynurenic acid | C10 H7 N O3 | 189.04191 | 4.773 | 76.9 |
| 38 | 5-[2-(3-Furyl)ethyl]-8a-(hydroxymethyl)-5,6-dimethyl-3,4,4a,5,6,7,8,8a-octahydro-1-naphthalenecarboxylic acid | C20 H28 O4 | 332.19759 | 13.417 | 76.7 |
| 39 | 2-Oxindole | C8 H7 N O | 133.05257 | 6.401 | 76.6 |
| 40 | 3-Coumaric acid | C9 H8 O3 | 164.04599 | 6.884 | 75.8 |
| 41 | Citric acid | C6 H8 O7 | 192.02566 | 1.326 | 75.7 |
| 42 | Gibberellin A7 | C19 H22 O5 | 330.14586 | 10.14 | 75.2 |
| 43 | Zearalenone | C18 H22 O5 | 300.13506 | 11.686 | 74.1 |
| 44 | 4-(4-Ethoxyphenyl)-4-oxobutanoic acid | C12 H14 O4 | 222.08809 | 10.034 | 73.8 |
| 45 | (2R)-5-Methoxy-2-methyl-2,3,8,9-tetrahydro-4H-furo[2,3-H]chromen-4-one | C13 H14 O4 | 234.08841 | 9.498 | 73.2 |
| 46 | D-(-)-Quinic acid | C7 H12 O6 | 192.06199 | 1.018 | 71.2 |
| 47 | 2-Hydroxyestradiol | C18 H24 O3 | 288.17165 | 11.647 | 70.9 |
| 48 | Columbianetin | C14 H14 O4 | 246.08826 | 10.893 | 69.8 |
| 49 | L-(+)-Tartaric acid | C4 H6 O6 | 150.01486 | 1.069 | 69 |
| 50 | 1-[2-(1,3-Benzodioxol-5-yl)-3-methyl-1-benzofuran-5-yl]-1,2-propanediol | C19 H18 O5 | 308.10355 | 12.349 | 69 |
| 51 | 2-Hydroxyhippuric acid | C9 H9 N O4 | 217.03545 | 0.9 | 68.7 |
| 52 | L-Pyroglutamic acid | C5 H7 N O3 | 129.04258 | 1.309 | 68.3 |
| 53 | Nalorphine | C19 H21 N O3 | 311.15114 | 12.251 | 66.3 |
| 54 | 6-Hydroxy-1-(hydroxymethyl)-5-{2-[2-(hydroxymethyl)-1-pyrrolidinyl]-2-oxoethyl}-1,4a-dimethyldecahydro-2-naphthalenyl phenylcarbamate | C27 H40 N2 O6 | 524.26076 | 9.328 | 65.5 |
| 55 | Gallic acid | C7 H6 O5 | 170.02004 | 1.082 | 64.7 |
| 56 | Jasmonic acid | C12 H18 O3 | 210.12432 | 11.278 | 64.6 |
| 57 | Gibberellin A4 | C19 H24 O5 | 332.16112 | 11.749 | 63.9 |
| 58 | Cortisone | C21 H28 O5 | 360.19242 | 9.795 | 63.6 |
| 59 | Hydrocortisone 17-butyrate | C25 H36 O6 | 432.24852 | 11.64 | 63.2 |
| 60 | 2-{(4S,5S,5aS,9aS)-4-Methoxy-6,6,9a-trimethyl-5-[(2E,4E,6E)-2,4,6-octatrienoyloxy]-1-oxo-1,3,4,5,5a,6,7,8,9,9a-decahydro-2H-benzo[E]isoindol-2-yl}pentanedioic acid | C29 H39 N O8 | 529.26932 | 10.145 | 62.4 |
| 61 | β-Asarone | C12 H16 O3 | 208.10936 | 7.939 | 62.3 |
| 62 | Butylparaben | C11 H14 O3 | 194.09319 | 9.936 | 61.8 |
| 63 | 4-Methylumbelliferone | C10 H8 O3 | 176.04673 | 8.932 | 61.5 |
| 64 | 4-Cyclopentylphenol | C11 H14 O | 162.10288 | 10.768 | 60.5 |

**Supplementary Table 3**

Shared components in JKZP aqueous extract, and JKZP-containing serum

| **No.** | **Name** | **Formula** | **Molecular Weight** |
| --- | --- | --- | --- |
| 1 | 1,2-Dipalmitoylphosphatidylglycerol | C38 H75 O10 P | 744.49148 |
| 2 | **Senkyunolide H** | C12 H16 O4 | 206.09315 |
| 3 | **Cryptotanshinone** | C19 H20 O3 | 296.13995 |
| 4 | 9-Oxo-10(E),12(E)-octadecadienoic acid | C18 H30 O3 | 294.21873 |
| 5 | **Loganin** | C17 H26 O10 | 407.17848 |
| 6 | 2,3,4,9-Tetrahydro-1H-β-carboline-3-carboxylic acid | C12 H12 N2 O2 | 216.08895 |
| 7 | PEG n5 | C10 H22 O6 | 238.14138 |
| 8 | PEG n10 | C20 H42 O11 | 458.27096 |
| 9 | Sorbic acid | C6 H8 O2 | 112.05263 |
| 10 | 3-Methoxycinnamic acid | C10 H10 O3 | 160.05185 |
| 11 | 3,5-di-tert-Butyl-4-hydroxybenzaldehyde | C15 H22 O2 | 234.16122 |
| 12 | PEG n12 | C24 H50 O13 | 546.32337 |
| 13 | PEG n11 | C22 H46 O12 | 502.29694 |
| 14 | Asiatic acid | C30 H48 O5 | 488.34883 |
| 15 | PEG n16 | C32 H66 O17 | 739.45365 |
| 16 | **Ferulic acid** | C10 H10 O4 | 194.05691 |
| 17 | 16α-Hydroxyestrone | C18 H22 O3 | 286.15632 |
| 18 | 4-Methoxycinnamic acid | C10 H10 O3 | 178.06228 |
| 19 | 8-Iso-15-keto-prostaglandin-F2β | C20 H32 O5 | 334.21365 |
| 20 | PEG n13 | C26 H54 O14 | 590.34903 |
| 21 | 4-Hydroxymandelic acid | C8 H8 O4 | 168.04072 |
| 22 | PEG n15 | C30 H62 O16 | 678.40164 |
| 23 | PEG n14 | C28 H58 O15 | 651.40184 |
| 24 | PPG n8 | C24 H50 O9 | 482.34382 |
| 25 | α-Estradiol | C18 H24 O2 | 272.177 |
| 26 | 6-Methylquinoline | C10 H9 N | 143.07297 |
| 27 | Phenylacetylglycine | C10 H11 N O3 | 193.07326 |
| 28 | Flurandrenolide | C24 H33 F O6 | 414.24523 |
| 29 | 6:2 Fluorinated telomer sulfonate | C8 H5 F13 O3 S | 427.97373 |
| 30 | PPG n5 | C15 H32 O6 | 308.21893 |
| 31 | Kynurenic acid | C10 H7 N O3 | 189.04191 |
| 32 | 5-[2-(3-Furyl)ethyl]-8a-(hydroxymethyl)-5,6-dimethyl-3,4,4a,5,6,7,8,8a-octahydro-1-naphthalenecarboxylic acid | C20 H28 O4 | 332.19759 |
| 33 | 3-Coumaric acid | C9 H8 O3 | 164.04599 |
| 34 | Citric acid | C6 H8 O7 | 192.02566 |
| 35 | Gibberellin A7 | C19 H22 O5 | 330.14586 |
| 36 | Zearalenone | C18 H22 O5 | 300.13506 |
| 37 | 4-(4-Ethoxyphenyl)-4-oxobutanoic acid | C12 H14 O4 | 222.08809 |
| 38 | (2R)-5-Methoxy-2-methyl-2,3,8,9-tetrahydro-4H-furo[2,3-H]chromen-4-one | C13 H14 O4 | 234.08841 |
| 39 | D-(-)-Quinic acid | C7 H12 O6 | 192.06199 |
| 40 | 2-Hydroxyestradiol | C18 H24 O3 | 288.17165 |
| 41 | Columbianetin | C14 H14 O4 | 246.08826 |
| 42 | L-(+)-Tartaric acid | C4 H6 O6 | 150.01486 |
| 43 | 1-[2-(1,3-Benzodioxol-5-yl)-3-methyl-1-benzofuran-5-yl]-1,2-propanediol | C19 H18 O5 | 308.10355 |
| 44 | 2-Hydroxyhippuric acid | C9 H9 N O4 | 217.03545 |
| 45 | L-Pyroglutamic acid | C5 H7 N O3 | 129.04258 |
| 46 | Nalorphine | C19 H21 N O3 | 311.15114 |
| 47 | Gallic acid | C7 H6 O5 | 170.02004 |
| 48 | Jasmonic acid | C12 H18 O3 | 210.12432 |
| 49 | Gibberellin A4 | C19 H24 O5 | 332.16112 |
| 50 | **β-Asarone** | C12 H16 O3 | 208.10936 |
| 51 | Butylparaben | C11 H14 O3 | 194.09319 |
| 52 | 4-Methylumbelliferone | C10 H8 O3 | 176.04673 |
| 53 | 4-Cyclopentylphenol | C11 H14 O | 162.10288 |
